# Dynapenic abdominal obesity and elevated risk of multidimensional multimorbidity across physical, psychological, and cognitive domains: evidence from longitudinal cohorts

**DOI:** 10.1265/ehpm.26-00041

**Published:** 2026-05-23

**Authors:** Xiqiang Zhang, Chenglu Jiang, Jiaming Li, Zhaoyi Jing, Yujia Song, Liang Peng, Baoyin Liu, Hua Meng

**Affiliations:** 1 Capital Medical University, China-Japan Friendship Hospital, Beijing, China; 2 Chinese Academy of Medical Sciences & Peking Union Medical College, China-Japan Friendship Hospital, Beijing, China; 3 Beijing Sport University, Beijing, China; 4 The First Clinical College, Shandong University of Traditional Chinese Medicine, Jinan, China; 5 China-Japan Friendship Hospital, Beijing, China; 6 China-Japan Friendship Hospital, Capital Medical University, Beijing, China

**Keywords:** Dynapenic abdominal obesity, Multidimensional multimorbidity, Physical, psychological and cognitive health, Longitudinal analysis, Risk factor

## Abstract

**Background:**

The long-term impact of dynapenic abdominal obesity (D/AO)—the coexistence of muscle weakness and central adiposity—on physical, psychological, and cognitive multidimensional multimorbidity (mMM) remains unclear.

**Methods:**

We analyzed longitudinal data from 8,311 adults aged ≥45 [China Health and Retirement Longitudinal Study (CHARLS), n = 4,887; Health and Retirement Study (HRS), n = 3,424] without baseline mMM (i.e., having at most a single-domain impairment), followed biennially for up to 4 years. To account for cross-cultural heterogeneity, analyses were conducted independently per cohort. Three mMM patterns—physical-psychological (PP-MM), physical-cognitive (PC-MM), and physical-psychological-cognitive (PPC-MM)—were constructed using chronic diseases, depressive symptoms, and cognitive tests. Multivariate regression assessed the risk and trajectory of mMM associated with D/AO, and continuous-time Markov multi-state models were utilized to estimate transition probabilities.

**Results:**

In both the CHARLS and HRS cohorts, D/AO was significantly associated with an increased risk of all three specific mMM patterns compared to the non-dynapenic/non-abdominal obesity group, with the strongest association observed for PPC-MM [OR = 2.475 (1.100–5.002) in CHARLS; OR = 5.712 (2.009–14.882) in HRS]. Longitudinal analysis revealed that D/AO was associated with a higher likelihood of entering “incident/worsening” trajectories (RRR = 2.001, P = 0.014 in CHARLS; RRR = 2.507, P = 0.013 in HRS) and an elevated probability of progressing from PC-MM to complex PPC-MM. Notably, age significantly modified these associations, with risk magnitudes markedly higher among individuals aged <65 years (e.g., PP-MM in HRS: OR = 6.98, 95% CI: 1.85–26.31; PC-MM in CHARLS: OR = 4.39, 95% CI: 1.95–9.89). Pooled analyses and multiple sensitivity tests confirmed the robustness of the D/AO “risk gradient effect” across diverse populations, without significant cohort interactions.

**Conclusion:**

D/AO is longitudinally associated with a higher incidence of mMM and less favorable health trajectories, particularly among individuals aged <65 years. These findings suggest that integrating muscle strength and abdominal adiposity assessments into primary care may offer potential value for early risk stratification. While the efficacy of targeted interventions warrants further verification in interventional studies, focusing on the D/AO phenotype provides a proactive perspective for mitigating the multidimensional multimorbidity burden in aging populations.

**Supplementary information:**

The online version contains supplementary material available at https://doi.org/10.1265/ehpm.26-00041.

## 1. Introduction

With the accelerating global ageing process [1], between 55% and 98% of older individuals present with at least two persistent conditions [2]. This pattern has become increasingly common in modern societies and represents an important clinical challenge, already affecting nearly one-fifth of adults even in low-income countries [3]. Notably, physical ailments are frequently intertwined with psychological distress and cognitive impairments [4, 5]. Evidence indicates that each additional chronic physical condition carries a 45% higher probability of depression [6], while comorbid states significantly reduce the likelihood of cognitive recovery [7]. Against the backdrop of pervasive urbanization and food industry globalization, a distinctive epidemiological transition, characterized by ‘multidimensional multimorbidity (mMM)’ spanning physical, psychological, and cognitive domains, is emerging [3]. With prevalence estimates ranging from 8.1% to 33.9% [8], individuals afflicted by mMM endure functional decline, premature mortality, and substantial medical expenditures, collectively imposing formidable pressure on global healthcare systems.

Against this backdrop, the coexistence of dynapenia and abdominal obesity—two aging-related health conditions—has garnered extensive attention [9, 10]. Dynapenia, characterized by the age-associated decline in muscle strength (maximal voluntary contraction), fundamentally reflects a progressive deterioration in neuromuscular function [11]. This impairment in individual functional activity [11], compounded by age-related reductions in metabolic rate and muscle-derived factors (myokines), favors the preferential accumulation of visceral fat [12]. Abdominal obesity has been highlighted as more detrimental than overall obesity [13]. It can induce reductions in muscle strength or mass mediated by diverse mechanisms, including chronic low-grade inflammation, disordered fatty acid metabolism, insulin resistance and oxidative stress [14–16]. This composite phenotype, termed “dynapenic abdominal obesity (D/AO),” may exert a systemic impact on the organism via the “neuro-immune-metabolic axis”. Pro-inflammatory cytokines (e.g., IL-6, TNF-α) released from abdominal fat can cross the blood-brain barrier to trigger neuroinflammation, directly compromising the hippocampus and prefrontal cortex, thereby precipitating cognitive decline and affective disorders [17]. Concurrently, diminished myokine secretion resulting from muscle weakness, alongside hypothalamic-pituitary-adrenal (HPA) axis dysregulation, further undermines the body’s resilience to stress and metabolic regulatory capacity [18]. These interconnected pathways may contribute to the development of mMM across physical, psychological, and cognitive domains. Although evidence suggests that D/AO significantly elevates the risk of osteoarthritis [19], cardiovascular disease [20], metabolic syndrome [21], depression [11], and cognitive impairment [22]—exceeding the risks associated with obesity or low muscle strength alone [23]—most existing studies have examined these two exposures or health outcomes in isolation [7]. This reductionist approach has left a substantial gap in understanding whether D/AO is associated with systemic deterioration of health across multiple dimensions.

Given that the management of multimorbidity is undergoing a paradigm shift—moving beyond a conventional disease-specific framework toward a patient-centered approach—the identification of critical upstream predictors has become paramount [24]. Leveraging two nationally representative prospective cohorts from China and the United States, this study investigates the longitudinal impact of D/AO on mMM. Our primary objective is to determine whether baseline D/AO is associated with an elevated incident risk of three specific mMM pattern: physical-psychological multimorbidity (PP-MM), physical-cognitive multimorbidity (PC-MM), and physical-psychological-cognitive multimorbidity (PPC-MM). Secondarily, we explore dynamic disease trajectories to elucidate how D/AO accelerates the transition from a healthy or single-morbidity state to complex multimorbidity. These findings aim to provide an evidence-based foundation for contemporary multimorbidity prevention and management (the relevant conceptual framework is presented in Supplementary Fig. 1).

## 2. Methods

### 2.1 Data sources and study population

**Fig. 1:**
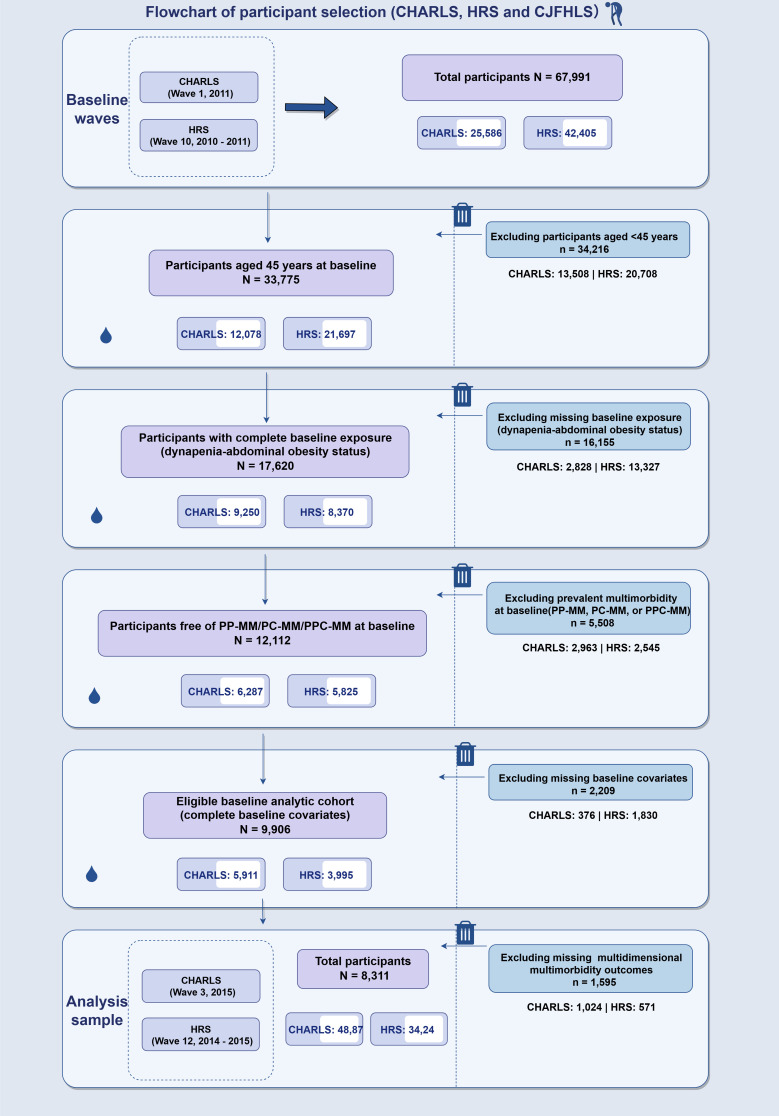
Participant selection flowchart in the CHARLS and HRS cohorts. CHARLS, China Health and Retirement Longitudinal Study; HRS, Health and Retirement Study; D/AO, dynapenic abdominal obesity; PP-MM, physical–psychological multimorbidity; PC-MM, physical–cognitive multimorbidity; PPC-MM, physical–psychological–cognitive multimorbidity.

This study utilized data from two international longitudinal aging cohorts: the China Health and Retirement Longitudinal Study (CHARLS) and the Health and Retirement Study (HRS). Both studies employed a multistage, stratified probability sampling framework (Supplementary Materials). Baseline data, corresponding to the initial exposure assessment, were derived from Wave 1 (2011) of CHARLS and Wave 10 (2010–2011) of HRS. The first follow-up data were collected from Wave 2 (2013) of CHARLS and Wave 11 (2012–2013) of HRS to assess outcome incidence and initial trajectory changes. Subsequent waves tracked the progression of outcomes until the final follow-up, specifically Wave 3 (2015) for CHARLS and Wave 12 (2014–2015) for HRS. Figure 1 illustrates the participant selection process. Using a complete-case approach, participants aged ≥45 years were screened, and those with missing data on exposures, outcomes, or any covariates were excluded. Crucially, to prospectively evaluate the incidence of mMM, individuals who already had PP-MM, PC-MM or PPC-MM at baseline were strictly excluded. In other words, the included participants possessed a maximum of a single-domain health impairment at baseline, ensuring that any mMM observed during the follow-up period represented true incident events. Ultimately, 8,311 eligible participants were included in the final analysis.

### 2.2 Dynapenia-abdominal obesity status

Muscle strength was quantified by handgrip dynamometry. This metric was selected as the key indicator of individual neuromuscular function, based on a substantial body of evidence demonstrating that muscle strength is a more robust predictor of adverse health outcomes—including falls, functional decline, and mortality—compared to muscle mass. Subjects underwent two trials bilaterally, and the peak reading across the four attempts was utilized. In the CHARLS cohort, dynapenia was defined as handgrip strength <28 kg for men and <18 kg for women, consistent with the 2019 Consensus Update from the Asian Working Group for Sarcopenia (AWGS 2019). Conversely, for the HRS cohort, dynapenia was identified based on the Foundation for the National Institutes of Health (FNIH) Sarcopenia Project, with sex-specific cut-off values of <26 kg and <16 kg, respectively. Central obesity was determined via waist circumference. Measurements were obtained in duplicate while participants stood upright, employing a non-stretchable tape positioned midway between the inferior costal margin and the iliac crest [25]. Abdominal obesity was defined by sex-specific waist circumference thresholds: ≥90 cm for men and ≥85 cm for women in the CHARLS cohort, and >102 cm and >88 cm, respectively, in the HRS cohort. Combining dynapenia and abdominal obesity, participants were classified into four mutually exclusive categories: non-dynapenic/non-abdominal obesity (ND/NAO); non-dynapenic/abdominal obesity (ND/AO); dynapenic/non-abdominal obesity (D/NAO); D/AO.

### 2.3 Study endpoints: multidimensional multimorbidity

Physical morbidity was identified based on self-reported physician diagnoses and relevant clinical criteria (Supplementary Table 1). In the CHARLS cohort, participants were classified as having physical morbidity if they reported a physician diagnosis of at least one of the following seven conditions: cancer, stroke, diabetes, chronic pulmonary disease, hypertension, arthritis and heart disease [8]; For the HRS cohort, the diagnostic criteria further integrated self-reported medical history, current medication use (e.g., antihypertensive, hypoglycemic, or cardiovascular drugs), and objective field measurements of blood pressure (systolic blood pressure ≥140 mmHg or diastolic blood pressure ≥90 mmHg).

Psychological status was evaluated via the Center for Epidemiologic Studies Depression Scale (CES-D), which has shown good validity in previous studies [26]. In addition, participants in both cohorts were asked whether they had ever been told that they had affective, emotional, nervous, or psychiatric problems.

Cognitive performance was assessed using domain-specific tests, including executive function (serial 7s subtraction test), episodic memory (immediate and delayed recall) and orientation (time, place and person). For each cognitive domain, age-specific mean scores were calculated separately for eight age groups: 45–49, 50–54, 55–59, 60–64, 65–69, 70–74, 75–79 and ≥80 years. A threshold of 1.5 standard deviations (SD) below the age-specific mean in a minimum of one cognitive domain was utilized to define cognitive impairment. In addition to test-based assessment, self-reported cognition-related conditions were also considered. In CHARLS, participants reported whether they had memory-related problems, whereas in HRS self-reported information included memory-related problems, dementia, and Alzheimer’s disease. This combined strategy was intended to improve sensitivity to clinically relevant cognitive impairment in large population-based cohorts.

In the physical domain, multimorbidity components were defined using self-reported physician-diagnosed chronic conditions, following common practice in large ageing cohorts. In contrast, the psychological and cognitive domains were defined using two sources of information: validated screening-based criteria and self-reported mental/cognition-related conditions. Participants were considered to have psychological or cognitive impairment if they met the screening threshold or reported the corresponding condition. This approach was adopted because, in community-based surveys such as CHARLS and HRS, clinically diagnosed depression, mild cognitive impairment, and dementia are often under-ascertained, whereas combining standardized screening tools with self-reported information provides a more feasible and comparable way to capture clinically meaningful impairment at the population level. Therefore, the multidimensional multimorbidity construct in this study reflects the coexistence of diagnosed physical disease burden and screening-/self-report-defined psychological and cognitive impairment burden, rather than three strictly diagnosis-based disease domains. On this basis, we constructed three multidimensional multimorbidity patterns: PP-MM, PC-MM, and PPC-MM. Study endpoints were ascertained at each discrete follow-up wave. For participants free of a given mMM pattern at baseline, an incident endpoint was recorded when they simultaneously met the corresponding domain-specific criteria for PP-MM, PC-MM, or PPC-MM at a subsequent follow-up wave.

### 2.4 Covariates

Based on previous multimorbidity research, we included sociodemographic variables and lifestyle behaviors as covariates. Sociodemographic factors comprised age, sex, marital status and education. Lifestyle behaviors included physical activity, smoking habits and alcohol intake. Physical activity was categorized as ‘active’ if the participant engaged in a minimum of one session of moderate-to-vigorous exercise per week, and ‘inactive’ otherwise. The unified definitions of all covariates are detailed in Supplementary Table 1.

### 2.5 Statistical analysis

Continuous variables following a Gaussian distribution were expressed as mean (SD) and compared via Student’s t-tests. Conversely, non-normally distributed data were summarized as medians with interquartile ranges [M (P25, P75)] and analyzed using the Mann-Whitney U test. Categorical variables are summarised as frequency (percentage) and were assessed via χ^2^ tests or Fisher’s exact tests, as appropriate. Given the inherent clinical overlap and cumulative nature of mMM—whereby individuals with triple-domain impairment (PPC-MM) inherently meet the criteria for dual-domain impairment (PP-MM/PC-MM)—logistic regression models were fitted to examine the longitudinal associations between baseline exposure and follow-up outcomes. Using ND/NAO as the reference group, we estimated odds ratios (ORs) and 95% confidence intervals (CIs) to quantify the risk across these interconnected health domains. Three hierarchical models were fitted for each exposure-outcome association: Model 1 was unadjusted; Model 2 controlled for sociodemographic characteristics; and Model 3 further adjusted for lifestyle behaviors.

Additionally, tests for trend were performed by treating dynapenia-abdominal Obesity status as an ordered categorical variable along two exposure pathways: T1 (ND/NAO → ND/AO → D/AO) and T2 (ND/NAO → D/NAO → D/AO). We constructed three-wave outcome trajectories based on binary outcomes (0/1) and categorized them into five patterns: stable disease-free (000), stable diseased (111), incident/worsening (001/011), improvement/recovery (100/110), and fluctuation (010/101). Multinomial logistic regression models were fitted to estimate relative risk ratios (RRRs) and 95% CIs, stratified by baseline status (using ‘stable disease-free’ and ‘stable diseased’ as references for baseline-free and baseline-diseased participants, respectively). Furthermore, participants were classified into four mutually exclusive states at each wave: S0 (free of relevant multimorbidity), S1 (PP-MM only), S2 (PC-MM only), and S3 (PPC-MM, treated as an absorbing state). Continuous-time Markov multi-state models were employed to estimate state transition intensities and state occupancy probabilities at t = 2 and t = 4 years. We further introduced interaction terms (“exposure × potential effect modifier”) into the effect models and used likelihood ratio tests or Wald tests to evaluate the statistical significance of interactions. Subgroup analyses were then conducted to characterise the distribution of effect estimates across strata.

Multiple sensitivity analyses were performed to verify the robustness of the findings: 1) Modified Poisson regression (MPR) with robust variance estimators was employed to re-estimate the relative risks (RRs) of the associations between exposures and outcomes; 2) We applied an alternative inclusion strategy based on single multimorbidity dimensions: for each multimorbidity pattern, we constructed analysis samples by excluding individuals with that specific multimorbidity at baseline and requiring complete follow-up information on the same outcome, thereby modelling each pattern separately; 3) We performed multiple imputation by chained equations to address missing covariate data, generating five imputed datasets with 20 iterations each using the “mice” package and pooling parameter estimates across datasets; 4) Multinomial logistic regression was employed to assess the risk of D/AO across mutually exclusive outcome states; 5) We pooled data from the two cohorts and included an indicator variable (‘Group’) as a fixed effect in the models to control for potential confounding arising from inter-cohort heterogeneity. All analyses were conducted using R software (version 4.5.1). Two-sided P values <0.05 were considered statistically significant.

## 3. Results

### 3.1 Baseline characteristics of the study cohorts

Table 1 summarizes the baseline characteristics of participants across the two cohorts. The mean ages (SD) of participants in the CHARLS and HRS cohorts were 57.7 (8.9) and 64.9 (10.6) years, respectively. Women accounted for over 50% of participants across all cohorts, particularly in the HRS, and the majority of participants were married or partnered. However, educational attainment varied substantially: in CHARLS, over 85% of participants had an education level below high school, whereas HRS participants exhibited significantly higher educational levels compared to those in CHARLS. Regarding lifestyle factors, approximately 59.4% of HRS participants were physically active, standing in sharp contrast to only about 11% in CHARLS. Notably, a higher prevalence of current smoking was observed in the CHARLS, whereas alcohol consumption was more prevalent in the HRS. In terms of outcomes, the prevalence of PP-MM ranged from 7.9% to 15.8%, and PC-MM ranged from 12.3% to 12.4%. In contrast, the prevalence of PPC-MM was relatively low, ranging from 2.1% to 4.9%.

**Table 1:** Baseline characteristics of study participants across the two cohorts.

**Characteristics**	**Study Cohort**	

	**CHARLS** **(n = 4887)**	**HRS** **(n = 3424)**
**Age**	57.7(8.9)	64.9(10.6)
**Gender**		
male	2437(49.9)	1393(40.7)
female	2450(50.1)	2031(59.3)
**Marital**		
married/partnered	4419(90.4)	2388(69.7)
other	468(9.6)	1036(30.3)
**Education**		
low	4204(86.0)	401(11.7)
high	683(14.0)	3023(88.3)
**Smoking**		
non-smoker	2903(59.4)	1727(50.4)
current smoker	1563(32.0)	403(11.8)
ex-smoker	421(8.6)	1294(37.8)
**Drinking**		
never	3322(68.0)	1806(52.7)
<1 drink/week	717(14.7)	770(22.5)
≥1 drink/week	848(17.4)	848(24.8)
**Physical activity**		
inactive	4322(88.4)	1391(40.6)
active	565(11.6)	2033(59.4)
**Dynapenia-abdominal obesity**		
ND/NAO	2695(55.1)	1071(31.3)
D/NAO	158(3.2)	48(1.4)
ND/AO	1954(40.0)	2220(64.8)
D/AO	80(1.6)	85(2.5)
**PP-MM**		
no	4116(84.2)	3154(92.1)
yes	771(15.8)	270(7.9)
**PC-MM**		
no	4284(87.7)	3000(87.6)
yes	603(12.3)	424(12.4)
**PPC-MM**		
no	4649(95.1)	3351(97.9)
yes	238(4.9)	73(2.1)

Data are presented as mean (SD) for continuous variables and n (%) for categorical variables. Abbreviations: CHARLS, China Health and Retirement Longitudinal Study; HRS, Health and Retirement Study; CJFHLS, China-Japan Friendship Hospital Longitudinal Study; ND/NAO, non-dynapenia and non-abdominal obesity; D/NAO, dynapenia and non-abdominal obesity; ND/AO, non-dynapenia and abdominal obesity; D/AO, dynapenic abdominal obesity; PP-MM, physical-psychological multimorbidity; PC-MM, physical-cognitive multimorbidity; PPC-MM, physical-psychological-cognitive multimorbidity.

### 3.2 Impact of dynapenia-abdominal obesity categories on mMM risk

To reduce confounding from measured covariates, multivariable-adjusted models were employed; however, the observed associations should be interpreted within the context of observational data. In CHARLS, after full adjustment (Model 3), the D/AO group was significantly associated with PP-MM [OR = 1.827 (1.076–3.009), *P* = 0.021], PC-MM [OR = 2.343 (1.337–3.958), *P* = 0.002], and PPC-MM [OR = 2.475 (1.100–5.002), *P* = 0.018] compared to the ND/NAO reference group (Table 2). Notably, D/NAO showed a significant association only with PPC-MM [OR = 2.126 (1.094–3.838), *P* = 0.018]. In the HRS, D/AO was similarly associated with all three outcomes, exhibiting generally larger effect sizes than those observed in CHARLS: PP-MM (OR = 3.118, 95%CI: 1.580–5.877), PC-MM (OR = 2.015, 95%CI: 1.101–3.553), and PPC-MM (OR = 5.712, 95%CI: 2.009–14.882). Conversely, no significant associations were observed between D/NAO and any of the three outcomes after full adjustment.

**Table 2:** Multivariable-adjusted associations between dynapenia-abdominal obesity phenotypes and multidimensional multimorbidity subtypes.

**Cohort**	**Multidimensional Multimorbidity**					

	**PP-MM**		**PC-MM**		**PPC-MM**	
		
	**OR (95%CI)**	**P**	**OR (95%CI)**	**P**	**OR (95%CI)**	**P**
**CHARLS**						
**Model 1**						
ND/NAO	Ref		Ref		Ref	
D/NAO	1.171 (0.743, 1.778)	0.476	1.368 (0.853, 2.107)	0.173	2.011 (1.058, 3.528)	0.022*
ND/AO	1.226 (1.0449, 1.438)	0.012*	1.105 (0.924, 1.32)	0.271	1.223 (0.929, 1.607)	0.15
D/AO	2.399 (1.435, 3.887)	0.001**	2.546 (1.48, 4.207)	<0.001***	2.844 (1.297, 5.547)	0.004**
**Model 2**						
ND/NAO	Ref		Ref		Ref	
D/NAO	1.017 (0.639, 1.563)	0.941	1.369 (0.843, 2.139)	0.185	2.073 (1.068, 3.733)	0.021*
ND/AO	1.119 (0.950, 1.317)	0.177	1.01 (0.841, 1.212)	0.916	1.05 (0.793, 1.388)	0.733
D/AO	1.806 (1.065, 2.973)	0.023*	2.286 (1.305, 3.857)	0.003**	2.342 (1.043, 4.722)	0.026*
**Model 3**						
ND/NAO	Ref		Ref		Ref	
D/NAO	1.009 (0.633, 1.551)	0.97	1.391 (0.856, 2.176)	0.164	2.126 (1.094, 3.838)	0.018*
ND/AO	1.123 (0.953, 1.323)	0.166	1.01 (0.84, 1.213)	0.917	1.068 (0.805, 1.414)	0.648
D/AO	1.827 (1.076, 3.009)	0.021*	2.343 (1.337, 3.958)	0.002**	2.475 (1.1, 5.002)	0.018*
**HRS**						
**Model 1**						
ND/NAO	Ref		Ref		Ref	
D/NAO	1.164 (0.277, 3.312)	0.803	2.016 (0.896, 4.094)	0.067	2.543 (0.397, 9.174)	0.22
ND/AO	1.672 (1.244, 2.282)	0.001***	1.292 (1.026, 1.637)	0.031*	1.238 (0.727, 2.199)	0.447
D/AO	3.743 (1.96, 6.789)	<0.001***	2.515 (1.422, 4.271)	0.001***	5.25 (1.99, 12.454)	<0.001***
**Model 2**						
ND/NAO	Ref		Ref		Ref	
D/NAO	1.072 (0.252, 3.114)	0.910	1.834 (0.795, 3.839)	0.127	3.015 (0.458, 11.52)	0.158
ND/AO	1.56 (1.151, 2.144)	0.005**	1.183 (0.93, 1.513)	0.175	1.135 (0.655, 2.046)	0.662
D/AO	3.462 (1.771, 6.452)	<0.001***	2.27 (1.248, 3.976)	0.005**	6.165 (2.221, 15.615)	<0.001***
**Model 3**						
ND/NAO	Ref		Ref		Ref	
D/NAO	1.085 (0.254, 3.171)	0.896	1.899 (0.817, 4.009)	0.11	3.208 (0.486, 12.338)	0.137
ND/AO	1.553 (1.136, 2.153)	0.007**	1.128 (0.882, 1.452)	0.343	1.235 (0.702, 2.26)	0.476
D/AO	3.118 (1.58, 5.877)	0.001***	2.015 (1.101, 3.553)	0.018*	5.712 (2.009, 14.882)	0.001***

Data are presented as odds ratios (ORs) [95% CIs)] derived from multivariable logistic regression models. Model 1: unadjusted; Model 2: adjusted for age, sex, marital status, and education; Model 3: additionally adjusted for smoking, alcohol consumption, and physical activity. Abbreviations: CHARLS, China Health and Retirement Longitudinal Study; HRS, Health and Retirement Study; ND/NAO, non-dynapenia and non-abdominal obesity; D/NAO, dynapenia and non-abdominal obesity; ND/AO, non-dynapenia and abdominal obesity; D/AO, dynapenic abdominal obesity; PP-MM, physical-psychological multimorbidity; PC-MM, physical-cognitive multimorbidity; PPC-MM, physical-psychological-cognitive multimorbidity; Ref, reference group.

Supplementary Table 2 shows that in both CHARLS and the HRS, the risk of mMM generally exhibited an increasing trend with the accumulation of exposure. Specifically, in CHARLS, the significant trend along the T1 pathway (accumulating towards D/AO) was primarily observed for PC-MM (*P* for trend = 0.001) and PPC-MM (*P* for trend = 0.002), whereas the trend along the T2 pathway was more pronounced for PP-MM (*P* for trend = 0.035). In the HRS, both pathways showed relatively consistent increasing trends for PP-MM and PPC-MM; however, the trends for PC-MM were mostly marginal or non-significant.

### 3.3 Longitudinal trajectories and state transition patterns of mMM

**Fig. 2:**
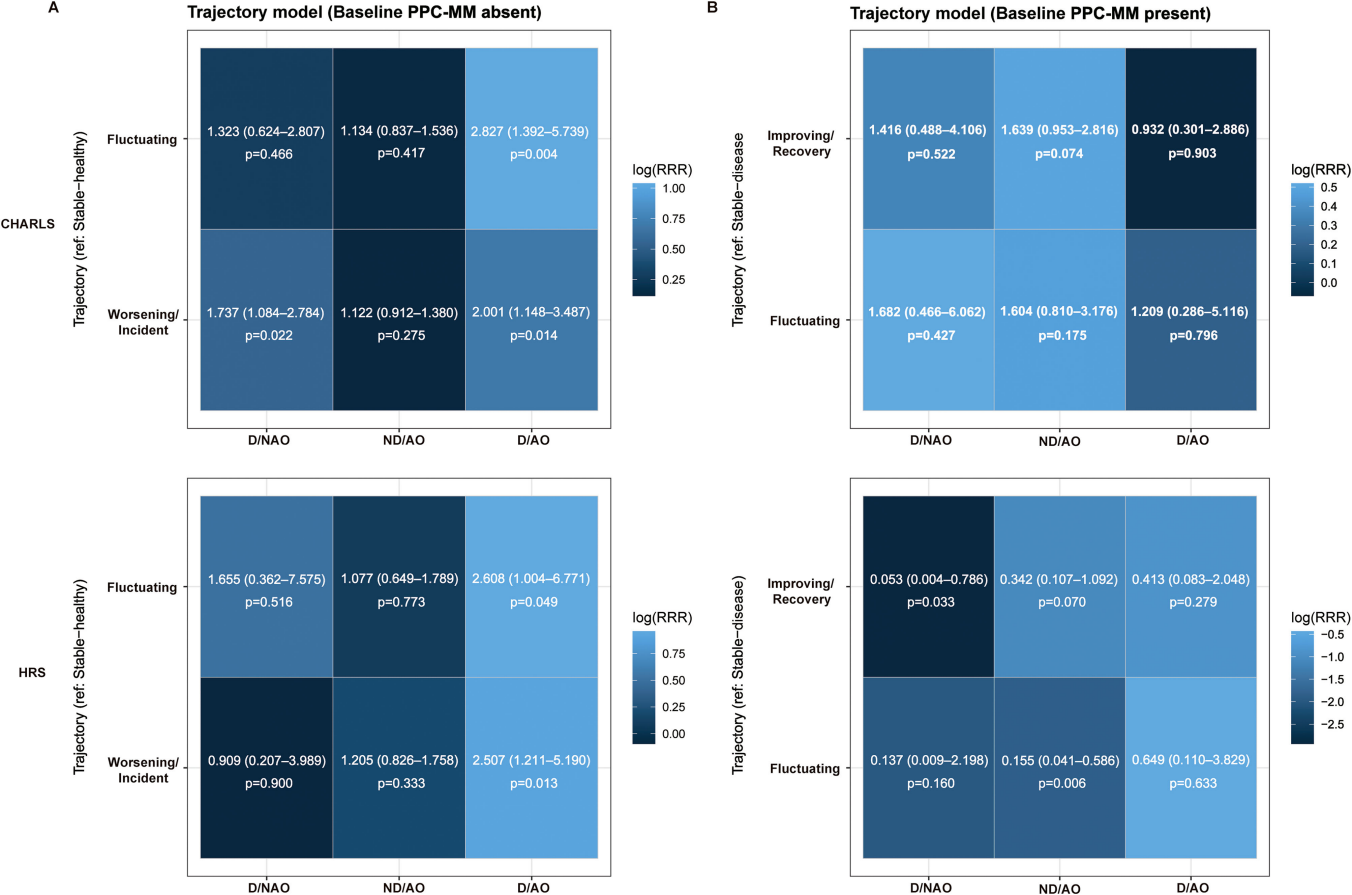
Associations of dynapenia-abdominal obesity phenotypes with longitudinal trajectories of PPC-MM stratified by baseline status. (A) Associations among participants free of PPC-MM at baseline. The reference trajectory group is “Stable-healthy.” The heatmaps display the relative risk of entering “Fluctuating” or “Worsening/Incident” trajectories compared to the stable-healthy group. (B) Associations among participants with existing PPC-MM at baseline. The reference trajectory group is “Stable-disease.” The heatmaps display the relative risk of entering “Improving/Recovery” or “Fluctuating” trajectories compared to the stable-disease group. In both panels, the color intensity represents the magnitude of the log-transformed Relative Risk Ratio (RRR), with darker blue indicating a stronger positive association. Values within cells denote the RRR (95% CI) and P-value derived from multivariable multinomial logistic regression models. Abbreviations: PPC-MM, physical-psychological-cognitive multimorbidity; CHARLS, China Health and Retirement Longitudinal Study; HRS, Health and Retirement Study; D/NAO, dynapenia and non-abdominal obesity; ND/AO, non-dynapenia and abdominal obesity; D/AO, dynapenic abdominal obesity.

Among participants free of PPC-MM at baseline, D/AO was associated with a higher likelihood of entering more adverse trajectories, a pattern that was consistent across all two cohorts (Fig. 2A). Specifically, regarding the “incident/worsening” trajectory, the RRRs were 2.001 (*P* = 0.014) in CHARLS and 2.507 (*P* = 0.013) in the HRS. A similar pattern was observed for the “fluctuation” trajectory. In contrast, among participants with pre-existing PPC-MM at baseline, no significant differences were generally observed between the D/AO group and the “improvement/recovery” or “fluctuation” trajectories (Fig. 2B). Furthermore, although D/NAO was associated with the “incident/worsening” trajectory in CHARLS (RRR = 1.737, *P* = 0.022), no consistent evidence was found in the HRS. Regarding participants free of PP-MM at baseline, D/AO was generally positively associated with adverse trajectories: while D/AO was associated with the “incident/worsening” trajectory in CHARLS (RRR = 1.762, *P* = 0.029), the effect sizes were further elevated in the HRS (Supplementary Fig. 2A). However, significant associations with the “fluctuation” trajectory were only observed in specific cohorts. Among those with pre-existing PP-MM at baseline, D/AO was associated with a reduced relative risk of entering the “improvement/recovery” trajectory only in the HRS cohort (RRR = 0.445, *P* = 0.035) (Supplementary Fig. 2B). Results for PC-MM were similar to those for PP-MM, except that the association between D/AO and the “incident/worsening” trajectory did not reach statistical significance in the HRS (Supplementary Fig. 3).

**Fig. 3:**
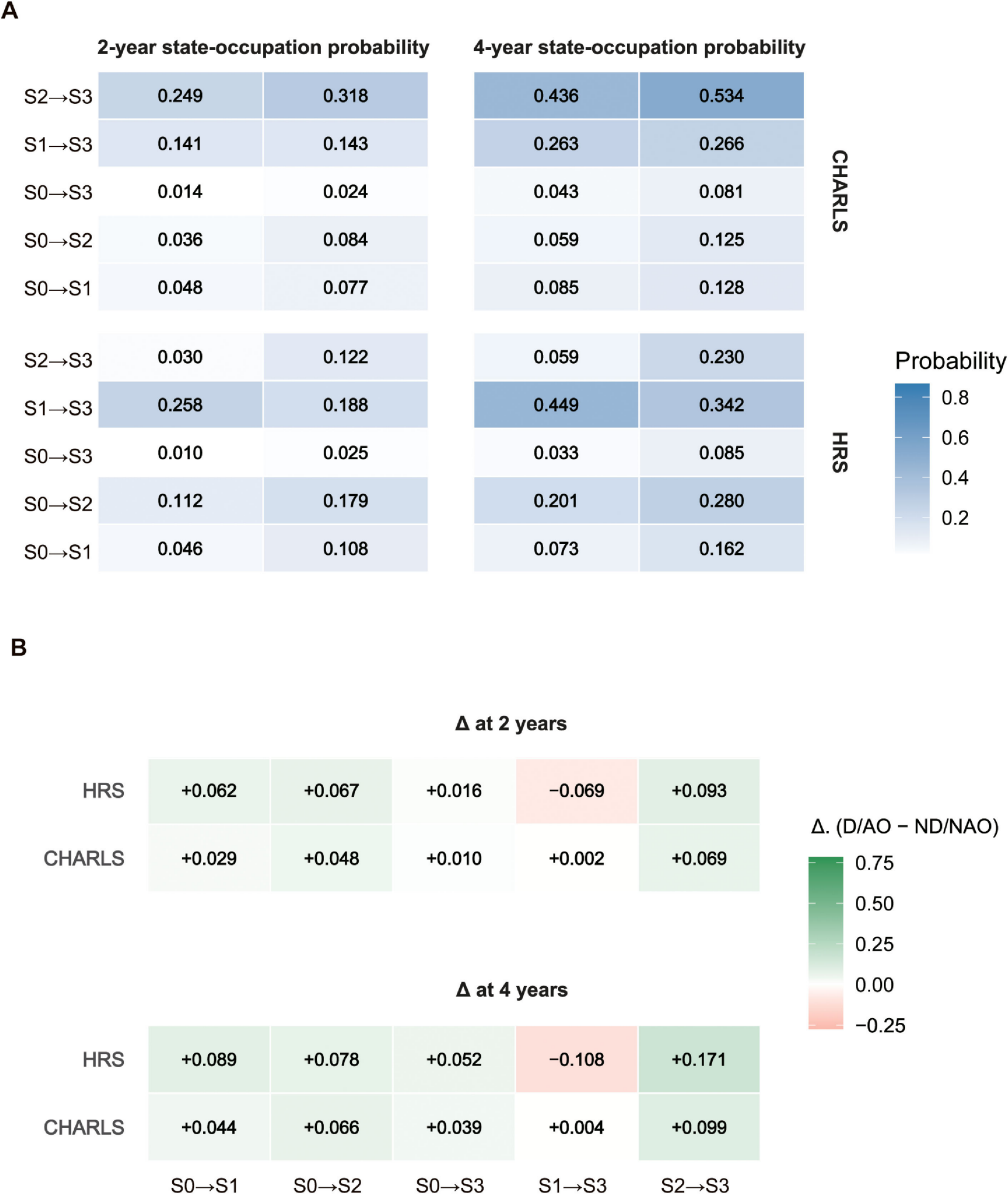
Multi-state modeling of transitions between multidimensional multimorbidity stages driven by D/AO across the two cohorts. The analysis utilizes continuous-time Markov models to estimate transition probabilities between four health states: S0 (free of relevant multimorbidity), S1 (PP-MM only), S2 (PC-MM only), and S3 (PPC-MM, treated as an absorbing state). (A) State-occupation probabilities at 2-year and 4-year intervals. (B) Differences (Δ) in transition probabilities. Heatmaps illustrate the absolute difference in state-occupation probabilities between the D/AO and ND/NAO groups. Green indicates a higher transition probability for the D/AO group (excess risk), while red indicates a lower probability. Abbreviations: CHARLS, China Health and Retirement Longitudinal Study; HRS, Health and Retirement Study; D/AO, dynapenic abdominal obesity; ND/NAO, non-dynapenia and non-abdominal obesity.

Based on the fitted continuous-time Markov multi-state models, Fig. 3 illustrates the transition probabilities between different multimorbidity states. From the perspective of disease incidence, regarding the 2-year state occupancy probabilities, D/AO participants exhibited a higher probability of transitioning from S0 to S2 compared to the ND/NAO group (CHARLS: 0.084; HRS: 0.179) (Fig. 3A); this finding was further supported by the differences in transition probabilities (Fig. 3B). Regarding the 4-year state occupancy probabilities, D/AO individuals in CHARLS (Δ = +0.066) remained more prone to incident PC-MM; however, distinct patterns emerged in the other cohorts, with HRS participants being more prone to incident PP-MM (Δ = +0.089) (Fig. 3B). From the perspective of disease progression, the transition trend from S2 to S3 was generally the most prominent across all cohorts (Fig. 3). Overall, individuals with D/AO showed higher estimated probabilities of entering the PC-MM state and of progressing from PC-MM to PPC-MM. The sample distribution of the total analytic sample across transition channels between adjacent follow-up waves is shown in Supplementary Fig. 4.

### 3.4 Interaction and subgroup analyses of D/AO and mMM

**Fig. 4:**
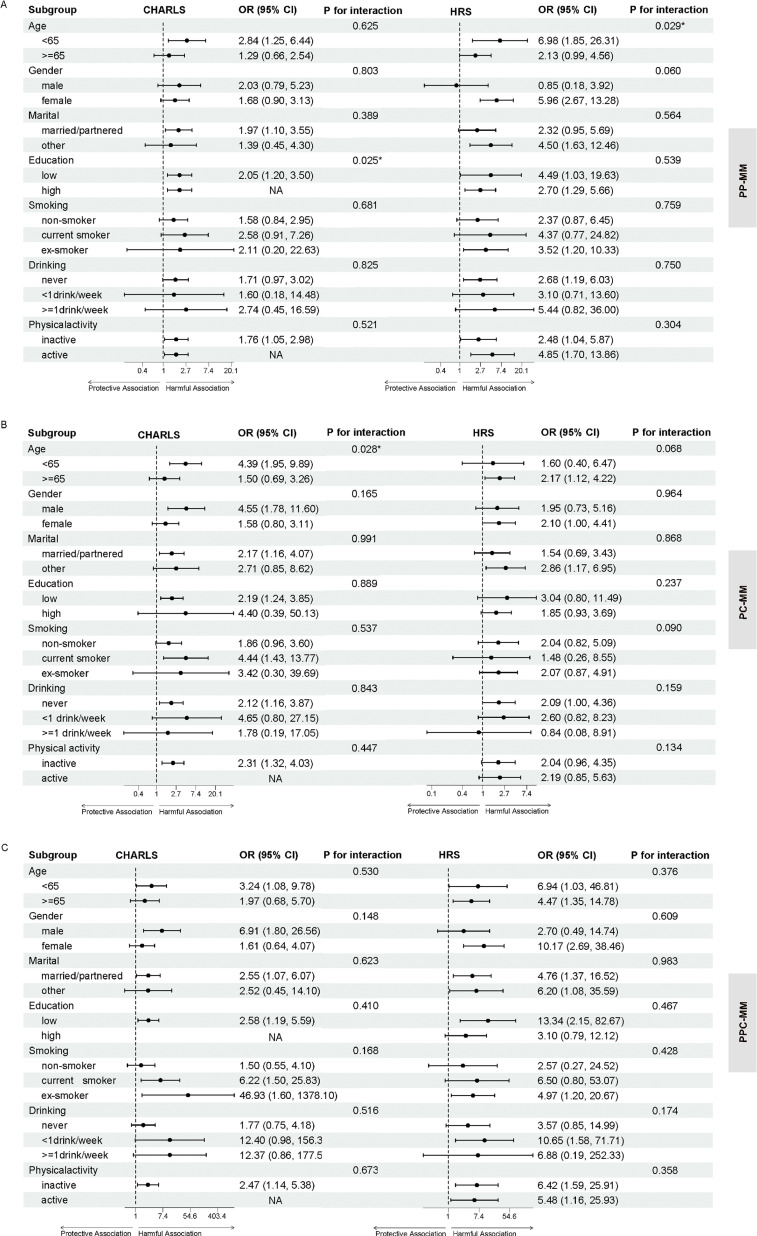
Subgroup and interaction analyses of the associations between D/AO and multidimensional multimorbidity. Forest plots display the multivariable-adjusted odds ratios (ORs) and 95% CIs derived from logistic regression models (Model 3) stratified by demographic and lifestyle factors. (A) Physical-psychological multimorbidity (PP-MM); (B) Physical-cognitive multimorbidity (PC-MM); and (C) Physical-psychological-cognitive multimorbidity (PPC-MM). P for interaction indicates the statistical significance of heterogeneity across strata. The vertical dashed line represents the null value (OR = 1); estimates to the right indicate a positive (“harmful”) association. Abbreviations: CHARLS, China Health and Retirement Longitudinal Study; HRS, Health and Retirement Study; D/AO, dynapenic abdominal obesity.

As shown in Fig. 4, the detrimental association between D/AO and increased mMM risk remained consistent across most subgroups. In the interaction tests, age and education level exhibited significant effect modifications. Specifically, in the HRS cohort, age significantly modified the association between D/AO and PP-MM (P for interaction = 0.029), with the risk magnitude being notably higher in individuals aged <65 years (OR = 6.98, 95% CI: 1.85–26.31). Similarly, in the CHARLS cohort, the association between D/AO and PC-MM was more pronounced among those aged <65 years (OR = 4.39, 95% CI: 1.95–9.89; P for interaction = 0.028). Furthermore, education level showed a significant modifying effect on PP-MM risk in the CHARLS cohort (P for interaction = 0.025), indicating that the association between D/AO and incident PP-MM was particularly pronounced among populations with lower educational attainment (OR = 2.05, 95% CI: 1.20–3.50).

### 3.5 Sensitivity analyses

Sensitivity analyses using MPR confirmed the robustness of the main findings regarding mMM across all two cohorts (Supplementary Table 3). Applying the “single-dimension exclusion” strategy, the effect estimates (Supplementary Table 4) and the progressive risk trends (Supplementary Table 5) across all two cohorts remained consistent with the main analyses. After addressing missing covariates using multiple imputation by chained equations, the robustness of the primary findings was confirmed, with the associations between D/AO status and all three mMM outcomes remaining positive and materially unchanged (Supplementary Table 6). Specifically, within the D/AO group, the ORs for PP-MM, PC-MM, and PPC-MM were 1.867, 2.343, and 2.641, respectively, in the CHARLS cohort; in the HRS cohort, the corresponding ORs were 2.509, 2.313, and 4.869. Consistent with the primary results, effect sizes in the HRS cohort for PP-MM and PPC-MM were generally larger than those in CHARLS, whereas the effect size for PC-MM was slightly lower. Furthermore, multinomial logistic regression results demonstrated a distinct “risk gradient effect” for the D/AO phenotype, even when treating outcomes as mutually exclusive states. In CHARLS, D/AO conferred the highest risk for PPC-MM (relative risk ratio [RRR] = 3.158, P = 0.004), significantly exceeding that for PC-MM (RRR = 2.347, P = 0.022). This trend was even more pronounced in the HRS cohort, where the RRR for PPC-MM associated with D/AO reached 6.532 (P < 0.001) (Supplementary Table 7). After pooling the two cohorts and including a fixed ‘Group’ effect in the models, the D/AO status consistently emerged as the group with the highest risk for all two outcomes. Furthermore, no significant interaction was observed between group and cohort (*P* for trend > 0.05). (Supplementary Table 8).

## 4. Discussion

The central finding of this study is that baseline D/AO was consistently associated with higher subsequent risk of mMM across physical, psychological, and cognitive domains. Based on the international prospective CHARLS and HRS cohorts, our analyses suggest that baseline D/AO is associated with higher incidence of PP-MM, PC-MM, and PPC-MM, as well as less favorable disease trajectories over time. Additionally, multi-state Markov analyses elucidate the dynamic trajectories of disease evolution: individuals with D/AO not only exhibit a higher propensity for incident PC-MM but are also highly susceptible to rapid deterioration from a dual-domain state (PC-MM) into complex, triple-domain multimorbidity (PPC-MM). Overall, this study highlights the combined association of D/AO with mMM risk, providing robust longitudinal evidence for the early and proactive management of multimorbidity.

The observed association between D/AO and an elevated risk of mMM, as well as its progression across three dimensions, may be linked to the dysregulation of the ‘muscle-fat-metabolism’ axis and subsequent cross-system neuro-immune cascades. A plausible pathway may involve impaired metabolic homeostasis: excessive visceral fat accumulation is accompanied by increased secretion of pro-inflammatory cytokines (e.g., IL-6, TNF-α) and reduced production of beneficial adipokines [27], fostering a state of chronic low-grade inflammation [16] and insulin resistance [15]. Concurrently, dynapenia reflects impaired skeletal muscle glucose uptake and diminished mitochondrial oxidative function. The co-occurrence of these two abnormalities may be associated with a heightened metabolic vulnerability, potentially predisposing individuals to a faster accumulation of chronic physical conditions such as hyperglycemia, hyperlipidemia, and cardiovascular disease [28, 29]. Subsequently, such systemic metabolic decline may be associated with alterations in the highly susceptible central nervous system. Because the brain is exquisitely sensitive to declines in glucose uptake and oxidative function, this energy metabolism deficit directly compromises memory, attention, and executive functions. Furthermore, peripheral inflammatory mediators can induce neuroinflammation and impair neuroplasticity [17], particularly affecting key brain regions such as the hippocampus and prefrontal cortex [30]; chronic inflammatory burden and oxidative stress also contribute to cerebral microvascular pathology and chronic hypoperfusion, further accelerating cognitive decline [31, 32]. Notably, within the multidimensional deterioration trajectory, cognitive decline typically precedes the onset of psychological symptoms. The persistent release of pro-inflammatory cytokines from excessively accumulated visceral fat hyperactivates the hypothalamic-pituitary-adrenal (HPA) axis [18], resulting in the disruption of cortisol rhythms. This prolonged neuroendocrine dysfunction not only exacerbates neuroinflammation but also disrupts neurotransmitter balance [33]. Ultimately, as cognitive reserve depletes and adaptive capacity diminishes, these biological alterations may increase vulnerability to psychological disorders such as depression and anxiety, potentially leading to the manifestation of complex, triple-domain multimorbidity.

Furthermore, this cross-system detrimental impact may be bidirectional. In turn, persistent psychological stress and symptoms of depression or anxiety can impair immune regulation, exacerbate inflammation, and promote abdominal fat accumulation and muscle protein breakdown [18, 34]. Cognitive decline reduces an individual’s capacity for disease self-management and health decision-making, increasing the likelihood of reduced physical activity, sleep disturbances and unhealthy dietary patterns, which in turn promote the accumulation of physical diseases [35, 36]. At the same time, cognitive impairment weakens stress-coping and emotional regulation, increasing social withdrawal, loneliness and depressive symptoms [37]. Together, these processes may form an “amplifying loop”, where initial impairment in one dimension is associated with cross-system cascades that further exacerbate mMM. (Supplementary Fig. S1).

Traditionally, clinical practice has managed physical diseases, depression, or cognitive impairment as isolated endpoints within a single-disease framework. However, by integrating physical, psychological, and cognitive functions into a multidimensional multimorbidity framework, this study captures the long-term coupling among the disease spectrum, functional status, and brain health. Our longitudinal findings suggest that D/AO is not merely a concurrent condition of aging; rather, it functions as a primary predictor of a systemic cascade of deterioration across multiple organs and systems. This conceptual shift is pivotal: it underscores the need to substantially shift the prevention window forward—moving from the reactive management of complex, established multimorbidity to the proactive identification and intervention of the upstream D/AO risk [24].

Furthermore, interaction and subgroup analyses revealed that the detrimental association between D/AO and mMM was more pronounced among individuals aged <65 years. This finding suggests that individuals exhibiting an “early-onset frailty phenotype” may be experiencing a more aggressive pathophysiological state, or it may foreshadow a longer trajectory of disease accumulation. Consequently, this demographic should be prioritized for early screening and intensive intervention. Additionally, the significant modifying effect of low education observed in the CHARLS cohort may reflects the systemic disadvantages faced by socioeconomically vulnerable populations regarding health literacy, healthcare accessibility, and the capacity to maintain healthy lifestyles [38].

While the positive associations between D/AO and mMM were highly consistent across both nations, the magnitude of these effects varied significantly. Specifically, the risk of PP-MM observed in the HRS cohort (OR = 3.118) was substantially higher than that in CHARLS (OR = 1.827), with an even more pronounced discrepancy for PPC-MM (OR = 5.712 in HRS vs. 2.475 in CHARLS). Conversely, the effect size for PC-MM was higher in CHARLS than in HRS. Several factors may underpin these variations. First, the more advanced chronic disease management systems and superior healthcare accessibility in the U.S. may enable HRS participants to receive earlier and more accurate diagnoses of subtle psychological and cognitive shifts. Second, the proportion of participants with higher education in the HRS (88.3%) far exceeded that in CHARLS (14.0%). While higher education typically correlates with better self-management, the systemic deterioration represented by D/AO may exert a statistically more dramatic impact when it disrupts a baseline of relatively high functional health. Third, lifestyle profiles differed sharply, with 59.4% of HRS participants being physically active compared to only 11.6% in CHARLS. Furthermore, the HRS cohort exhibited higher alcohol consumption, whereas current smoking was more prevalent in CHARLS. These distinct exposure combinations likely modulate the synergistic intensity between D/AO and mental/cognitive health outcomes. Finally, ethnic-specific metabolic sensitivity to abdominal adiposity accumulation may also contribute to these differences [39]. These cross-cohort discrepancies may reflect differences in healthcare systems, risk exposure structures, and sociocultural contexts, and they underscore the importance of considering population-level context when interpreting the findings.

Extensive sensitivity analyses and the observed consistency across subgroups enhance the robustness of our findings; nevertheless, the potential for residual bias and unobserved heterogeneity remains: (1) Despite efforts to harmonize data, inherent differences remain across cohorts in cognitive assessment tools, sensitivity of depression scales, disease ascertainment methods and study designs. In addition, pooled analyses did not incorporate sampling weights, which may introduce systematic bias; (2) Multimorbidity was ascertained via self-report, which may introduce inherent information bias, including memory-related and social desirability constraints. Moreover, we only included seven physical conditions, which may underrepresent the actual burden of multimorbidity; (3) Although models adjusted for multiple factors, unmeasured confounders—including dietary patterns, sleep quality and access to care—may still influence the observed associations; (4) Direct measurements of muscle mass were unavailable in this large-scale cohort setting. Consequently, we could not differentiate isolated low muscle strength from low muscle strength accompanied by reduced muscle mass, nor could we distinguish skeletal muscle mass, intramuscular fat infiltration, or visceral from subcutaneous adiposity. These limitations may have constrained a more granular phenotypic characterization of D/AO; (5) Data on symptom severity, disease duration and subtypes of psychological and cognitive impairment were not available, limiting more granular stratified analyses of exposure-outcome relations; (6) Caution should be exercised when generalizing these findings to resource-limited settings; thus, future research involving more geographically and culturally diverse samples is warranted.

Building on these profound long-term impacts, the observational evidence from this study supports the potential utility of further evaluating handgrip strength and waist circumference as pragmatic markers in routine risk assessment. These measurements are low-cost, easy to perform, and particularly suitable for widespread implementation in resource-constrained primary care settings. Furthermore, for individuals already on a multimorbidity trajectory, conventional organ-specific clinical models may be insufficient to forestall the progression toward complex, triple-domain multimorbidity (PPC-MM). Conversely, implementing comprehensive interventions—including resistance exercise training, nutritional optimization, weight management, and emotion regulation—could theoretically help fundamentally disrupt the vicious cycle of neuro-immune-metabolic imbalance. However, it must be emphasized that as an observational cohort study, our findings cannot directly establish the causal interventional efficacy of these clinical measures. Future well-designed interventional studies and randomized controlled trials are urgently needed to rigorously verify whether routine screening combined with early multidisciplinary interventions can effectively halt or reverse the accumulation of multidimensional multimorbidity. At the public health level, integrating mMM across physical, psychological, and cognitive domains into surveillance frameworks may be worth considering. Prevention strategies may benefit from greater focus on midlife and younger-old populations (<65 years), especially in view of the subgroup findings. Future interdisciplinary collaboration and policy-level design remain necessary to explore how best to embed D/AO risk identification into existing primary care chronic disease management and social support systems.

## 5. Conclusion

Based on two nationally prospective aging cohorts, this study provides longitudinal evidence that D/AO is associated with a higher incidence of mMM across physical, psychological, and cognitive domains among middle-aged and older adults, with these associations being particularly pronounced among individuals aged <65 years. Furthermore, D/AO was associated with less favorable health trajectories during follow-up. Our findings suggest that the early identification of reduced muscle strength and abdominal adiposity in combination may have potential value for identifying individuals at higher risk of multimorbidity in primary care settings. Although the routine assessment of waist circumference and handgrip strength, alongside early multidimensional intervention strategies, warrant further evaluation in intervention studies, focusing on the early evolution of the D/AO phenotype may offer useful perspectives for multimorbidity risk stratification and prevention in the context of global aging.

## Abbreviations

mMM: multidimensional multimorbidity
D/AO: dynapenic abdominal obesity
CHARLS: China Health and Retirement Longitudinal Study
HRS: Health and Retirement Study
ND/NAO: non-dynapenic/non-abdominal obesity
ND/AO: non-dynapenic/abdominal obesity
D/NAO: dynapenic/non-abdominal obesity
CES-D: Center for Epidemiologic Studies Depression Scale
PP-MM: physical-psychological multimorbidity
PC-MM: physical-cognitive multimorbidity
PPC-MM: physical-psychological-cognitive multimorbidity
ORs: odds ratios
CIs: confidence intervals
RRRs: relative risk ratios
MPR: Modified Poisson regression
RRs: Relative risks
HPA: hypothalamic-pituitary-adrenal

## Supplementary information

**Additional file 1:** **Supplementary Figure 1.** Schematic diagram illustrating the associations between dynapenic abdominal obesity and multidimensional multimorbidity. The diagram outlines the integrated study hypothesis and key findings. (Top Left) The Exposure: Dynapenia (low muscle strength) and abdominal obesity (central adiposity) converge to form the D/AO phenotype, creating a vicious cycle of cumulative metabolic and functional burden. (Center) The Outcome: D/AO acts as a synergistic risk factor for the multidimensional multimorbidity (mMM) complex, encompassing physical comorbidity, psychological distress, and cognitive decline. (Top Right) Pathophysiological Mechanisms: The proposed neuro-immune-metabolic axis links D/AO to mMM. Key pathways include chronic inflammation (e.g., IL-6, TNF-α) released by inflamed adipose tissue, myokine deficiency, HPA axis dysregulation, neuroinflammation, and vascular oxidative stress, which collectively accelerate systemic frailty and cross-system deterioration. (Bottom Left) Study Context: The association was validated across two independent cohorts (CHARLS and HRS), showing a robust dose-response risk gradient, where the D/AO phenotype confers the highest risk for incident PPC-MM. (Bottom Right) Implications: The findings underscore the need for integrated interventions, including routine screening for muscle strength and waist circumference, combined with strength training, weight management, and mental health promotion to slow mMM accumulation. Abbreviations: D/AO, dynapenic abdominal obesity; mMM, multidimensional multimorbidity; HPA, hypothalamic-pituitary-adrenal.

**Additional file 2:** **Supplementary Figure 2.** Associations of dynapenia-abdominal obesity phenotypes with longitudinal trajectories of PP-MM stratified by baseline status. (A) Associations among participants free of PP-MM at baseline. The reference trajectory group is “Stable-healthy.” The heatmaps display the relative risk of entering “Fluctuating” or “Worsening/Incident” trajectories compared to the stable-healthy group. (B) Associations among participants with existing PP-MM at baseline. The reference trajectory group is “Stable-disease.” The heatmaps display the relative risk of entering “Improving/Recovery” or “Fluctuating” trajectories compared to the stable-disease group. In both panels, the color intensity represents the magnitude of the log-transformed Relative Risk Ratio (RRR), with darker blue indicating a stronger positive association. Values within cells denote the RRR (95% CI) and P-value derived from multivariable multinomial logistic regression models. Abbreviations: PP-MM, physical-psychological multimorbidity; CHARLS, China Health and Retirement Longitudinal Study; HRS, Health and Retirement Study; D/NAO, dynapenia and non-abdominal obesity; ND/AO, non-dynapenia and abdominal obesity; D/AO, dynapenic abdominal obesity.

**Additional file 3:** **Supplementary Figure 3.** Associations of dynapenia-abdominal obesity phenotypes with longitudinal trajectories of PC-MM stratified by baseline status. (A) Associations among participants free of PC-MM at baseline. The reference trajectory group is “Stable-healthy.” The heatmaps display the relative risk of entering “Fluctuating” or “Worsening/Incident” trajectories compared to the stable-healthy group. (B) Associations among participants with existing PC-MM at baseline. The reference trajectory group is “Stable-disease.” The heatmaps display the relative risk of entering “Improving/Recovery” or “Fluctuating” trajectories compared to the stable-disease group. In both panels, the color intensity represents the magnitude of the log-transformed Relative Risk Ratio (RRR), with darker blue indicating a stronger positive association. Values within cells denote the RRR (95% CI) and P-value derived from multivariable multinomial logistic regression models. Abbreviations: PC-MM, physical-cognitive multimorbidity; CHARLS, China Health and Retirement Longitudinal Study; HRS, Health and Retirement Study; D/NAO, dynapenia and non-abdominal obesity; ND/AO, non-dynapenia and abdominal obesity; D/AO, dynapenic abdominal obesity.

**Additional file 4:** **Supplementary Figure 4.** Observed frequency of transitions between mMM in the total analytic sample across adjacent follow-up waves. The matrices display the raw counts of all participants included in the analysis transitioning from a starting state (rows, “From state”) to a subsequent state (columns, “To state”) over specific time intervals (e.g., 2011–2013 and 2013–2015). Color intensity corresponds to the number of participants, with darker blue indicating higher frequencies. Diagonal cells represent individuals who remained in the same state (stability), while off-diagonal cells represent those who transitioned to a different state (worsening or improvement). Health states are defined as: S0, free of multimorbidity; S1, physical-psychological multimorbidity (PP-MM); S2, physical-cognitive multimorbidity (PC-MM); and S3, physical-psychological-cognitive multimorbidity (PPC-MM). Abbreviations: CHARLS, China Health and Retirement Longitudinal Study; HRS, Health and Retirement Study.

**Additional file 5:** **Supplementary Materials.**

**Additional file 6:** **Supplementary Table 1.** Harmonized definitions and measurement strategies for exposures, covariates and outcomes in the present study.

**Additional file 7:** **Supplementary Table 2.** Trends in multimorbidity risk across different phenotypic progression pathways of dynapenia-abdominal obesity.

**Additional file 8:** **Supplementary Table 3.** Modified Poisson regression analyses of dynapenia–abdominal obesity status in relation to multidimensional multimorbidity.

**Additional file 9:** **Supplementary Table 4.** Associations of dynapenia–abdominal obesity status with multidimensional multimorbidity under the single-dimension exclusion strategy.

**Additional file 10:** **Supplementary Table 5.** Trend analyses of the associations between exposure and multidimensional multimorbidity under the single-dimension exclusion strategy.

**Additional file 11:** **Supplementary Table 6.** Associations of dynapenia–abdominal obesity status with multidimensional multimorbidity after multiple imputation.

**Additional file 12:** **Supplementary Table 7.** Multinomial logistic regression for the associations between dynapenia-abdominal obesity phenotypes and mutually exclusive multidimensional multimorbidity.

**Additional file 13:** **Supplementary Table 8.** Pooled associations of dynapenic abdominal obesity status with multidimensional multimorbidity and assessment of cohort heterogeneity.

## Declarations

### Availability of data and materials

The datasets for CHARLS and HRS are publicly available for academic use via their respective official portals: https://charls.pku.edu.cn and https://hrs.isr.umich.edu. These data were accessed following standard registration and anonymization protocols. The data from the CJFHLS cohort are not publicly available due to institutional restrictions and participant privacy. However, de-identified data may be made available from the corresponding author upon reasonable request and subject to ethical approval.
