# Supplementary material for: Dynapenic abdominal obesity and elevated risk of multidimensional multimorbidity across physical, psychological, and cognitive domains: evidence from longitudinal cohorts
Source: Environ Health Prev Med. 2026 May 23;31:35. doi: 10.1265/ehpm.26-00041 (PMC13222744; doi:10.1265/ehpm.26-00041)

DYNAPENIC ABDOMINAL OBESITY (D/AO): CATALYST FOR MULTIDIMENSIONAL MULTIMORBIDITY

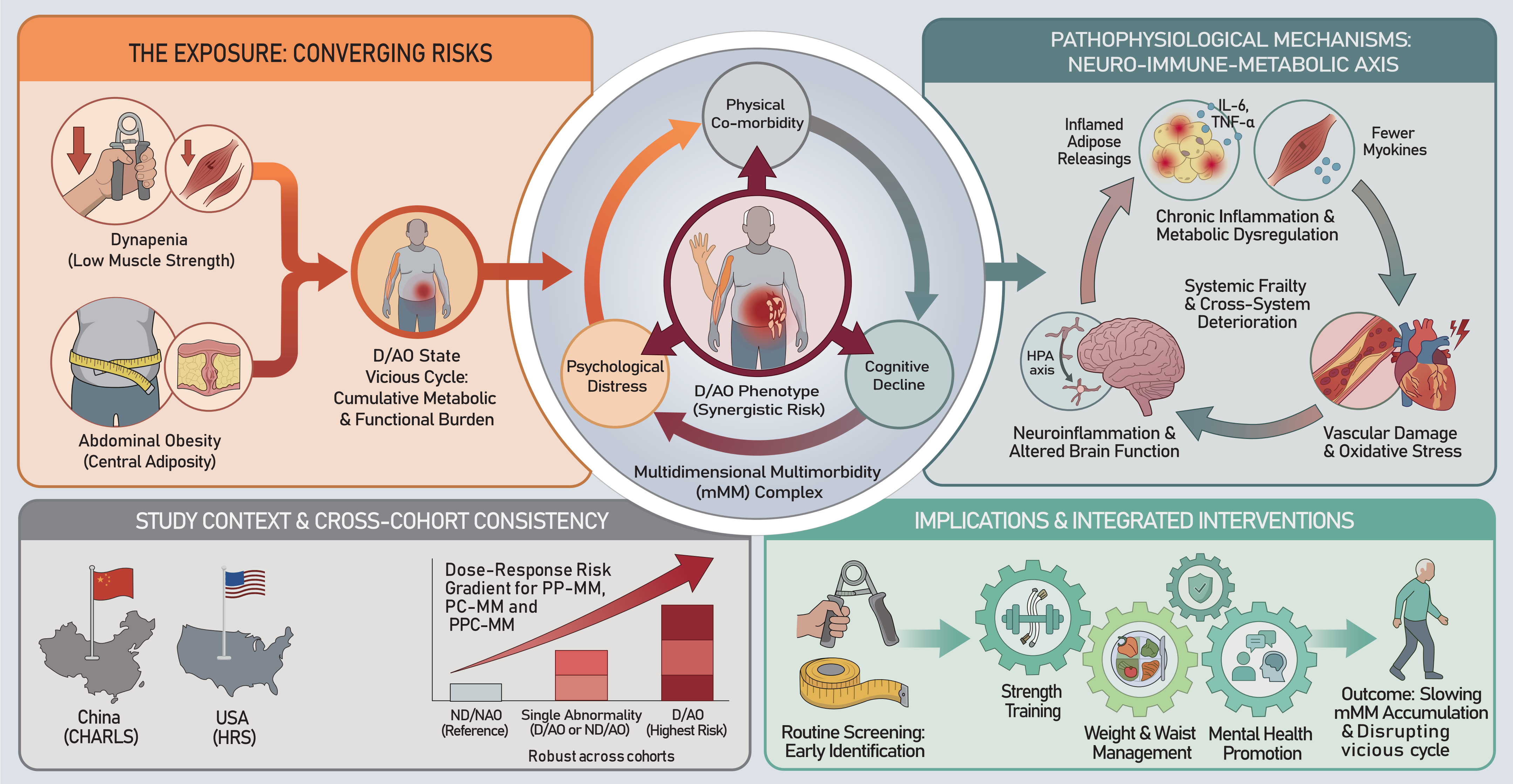

Supplement: Supplementary file 1 — Additional file 1: Supplementary Figure 1. Schematic diagram illustrating the associations between dynapenic abdominal obesity and multidimensional multimorbidity. The diagram outlines the integrated study hypothesis and key findings. (Top Left) The Exposure: Dynapenia (low muscle strength) and abdominal obesity (central adiposity) converge to form the D/AO phenotype, creating a vicious cycle of cumulative metabolic and functional burden. (Center) The Outcome: D/AO acts as a synergistic risk factor for the multidimensional multimorbidity (mMM) complex, encompassing physical comorbidity, psychological distress, and cognitive decline. (Top Right) Pathophysiological Mechanisms: The proposed neuro-immune-metabolic axis links D/AO to mMM. Key pathways include chronic inflammation (e.g., IL-6, TNF-α) released by inflamed adipose tissue, myokine deficiency, HPA axis dysregulation, neuroinflammation, and vascular oxidative stress, which collectively accelerate systemic frailty and cross-system deterioration. (Bottom Left) Study Context: The association was validated across two independent cohorts (CHARLS and HRS), showing a robust dose-response risk gradient, where the D/AO phenotype confers the highest risk for incident PPC-MM. (Bottom Right) Implications: The findings underscore the need for integrated interventions, including routine screening for muscle strength and waist circumference, combined with strength training, weight management, and mental health promotion to slow mMM accumulation. Abbreviations: D/AO, dynapenic abdominal obesity; mMM, multidimensional multimorbidity; HPA, hypothalamic-pituitary-adrenal. [file ehpm-31-035-s001.pdf]
