# Supplementary material for: Dynapenic abdominal obesity and elevated risk of multidimensional multimorbidity across physical, psychological, and cognitive domains: evidence from longitudinal cohorts
Source: Environ Health Prev Med. 2026 May 23;31:35. doi: 10.1265/ehpm.26-00041 (PMC13222744; doi:10.1265/ehpm.26-00041)
Supplement: Supplementary file 2 — Additional file 2: Supplementary Figure 2. Associations of dynapenia-abdominal obesity phenotypes with longitudinal trajectories of PP-MM stratified by baseline status. (A) Associations among participants free of PP-MM at baseline. The reference trajectory group is “Stable-healthy.” The heatmaps display the relative risk of entering “Fluctuating” or “Worsening/Incident” trajectories compared to the stable-healthy group. (B) Associations among participants with existing PP-MM at baseline. The reference trajectory group is “Stable-disease.” The heatmaps display the relative risk of entering “Improving/Recovery” or “Fluctuating” trajectories compared to the stable-disease group. In both panels, the color intensity represents the magnitude of the log-transformed Relative Risk Ratio (RRR), with darker blue indicating a stronger positive association. Values within cells denote the RRR (95% CI) and P-value derived from multivariable multinomial logistic regression models. Abbreviations: PP-MM, physical-psychological multimorbidity; CHARLS, China Health and Retirement Longitudinal Study; HRS, Health and Retirement Study; D/NAO, dynapenia and non-abdominal obesity; ND/AO, non-dynapenia and abdominal obesity; D/AO, dynapenic abdominal obesity. [file ehpm-31-035-s002.pdf]

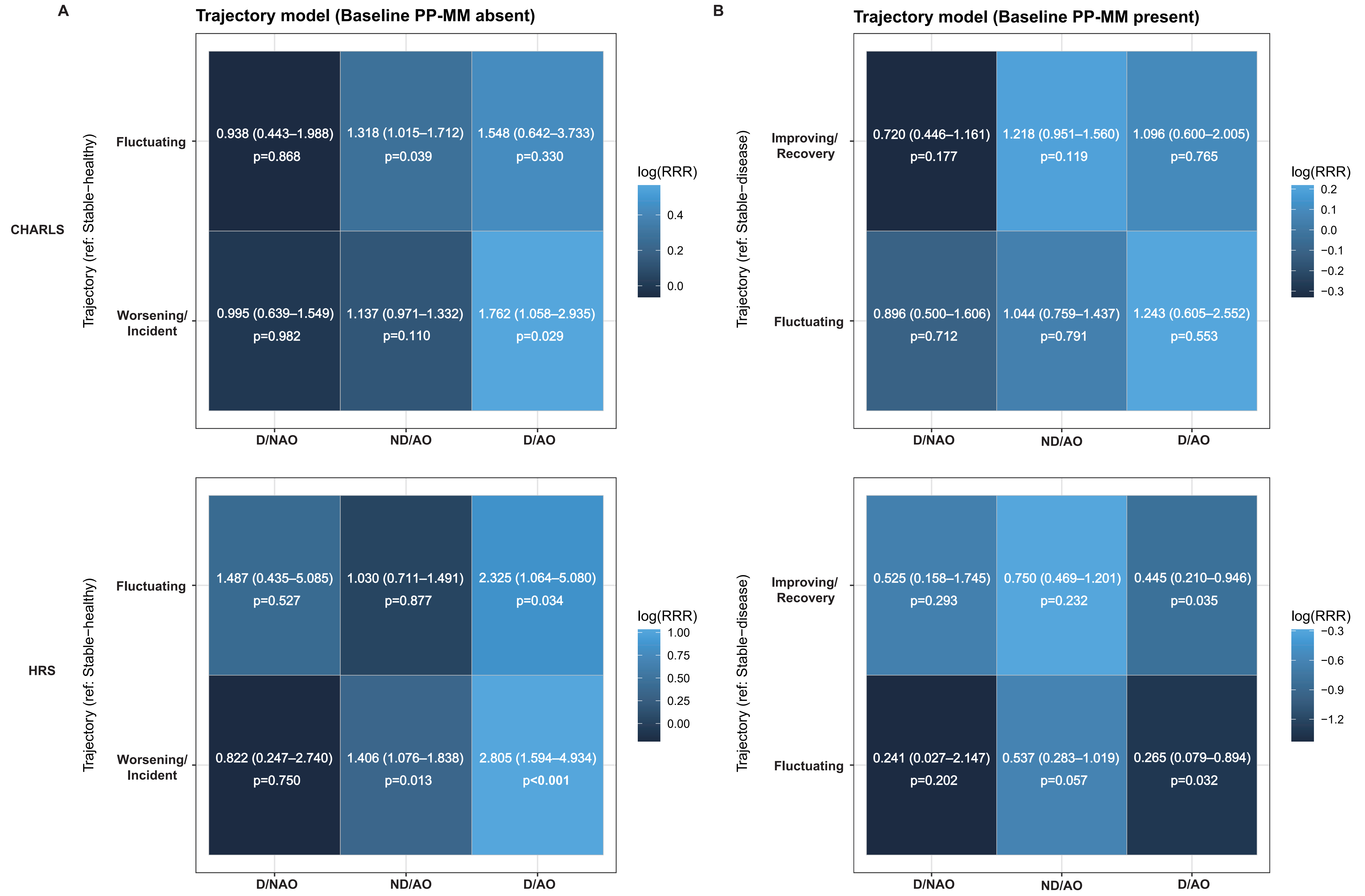
A
Trajectory model (Baseline PP-MM absent)
B
Trajectory model (Baseline PP-MM present)
CHARLS
HRS
Trajectory (ref: Stable−healthy)
Trajectory (ref: Stable−disease)
Fluctuating
Worsening/ Incident
Improving/ Recovery
D/NAO
ND/AO
D/AO
log(RRR)
0.938 (0.443–1.988) p=0.868
1.318 (1.015–1.712) p=0.039
1.548 (0.642–3.733) p=0.330
0.995 (0.639–1.549) p=0.982
1.137 (0.971–1.332) p=0.110
1.762 (1.058–2.935) p=0.029
0.720 (0.446–1.161) p=0.177
1.218 (0.951–1.560) p=0.119
1.096 (0.600–2.005) p=0.765
0.896 (0.500–1.606) p=0.712
1.044 (0.759–1.437) p=0.791
1.243 (0.605–2.552) p=0.553
1.487 (0.435–5.085) p=0.527
1.030 (0.711–1.491) p=0.877
2.325 (1.064–5.080) p=0.034
0.822 (0.247–2.740) p=0.750
1.406 (1.076–1.838) p=0.013
2.805 (1.594–4.934) p<0.001
0.525 (0.158–1.745) p=0.293
0.750 (0.469–1.201) p=0.232
0.445 (0.210–0.946) p=0.035
0.241 (0.027–2.147) p=0.202
0.537 (0.283–1.019) p=0.057
0.265 (0.079–0.894) p=0.032
0.4
0.2
0.0
0.2
0.1
0.0
−0.1
−0.2
−0.3
1.00
0.75
0.50
0.25
0.00
−0.3
−0.6
−0.9
−1.2
