# Supplementary material for: Dynapenic abdominal obesity and elevated risk of multidimensional multimorbidity across physical, psychological, and cognitive domains: evidence from longitudinal cohorts
Source: Environ Health Prev Med. 2026 May 23;31:35. doi: 10.1265/ehpm.26-00041 (PMC13222744; doi:10.1265/ehpm.26-00041)
Supplement: Supplementary file 3 — Additional file 3: Supplementary Figure 3. Associations of dynapenia-abdominal obesity phenotypes with longitudinal trajectories of PC-MM stratified by baseline status. (A) Associations among participants free of PC-MM at baseline. The reference trajectory group is “Stable-healthy.” The heatmaps display the relative risk of entering “Fluctuating” or “Worsening/Incident” trajectories compared to the stable-healthy group. (B) Associations among participants with existing PC-MM at baseline. The reference trajectory group is “Stable-disease.” The heatmaps display the relative risk of entering “Improving/Recovery” or “Fluctuating” trajectories compared to the stable-disease group. In both panels, the color intensity represents the magnitude of the log-transformed Relative Risk Ratio (RRR), with darker blue indicating a stronger positive association. Values within cells denote the RRR (95% CI) and P-value derived from multivariable multinomial logistic regression models. Abbreviations: PC-MM, physical-cognitive multimorbidity; CHARLS, China Health and Retirement Longitudinal Study; HRS, Health and Retirement Study; D/NAO, dynapenia and non-abdominal obesity; ND/AO, non-dynapenia and abdominal obesity; D/AO, dynapenic abdominal obesity. [file ehpm-31-035-s003.pdf]

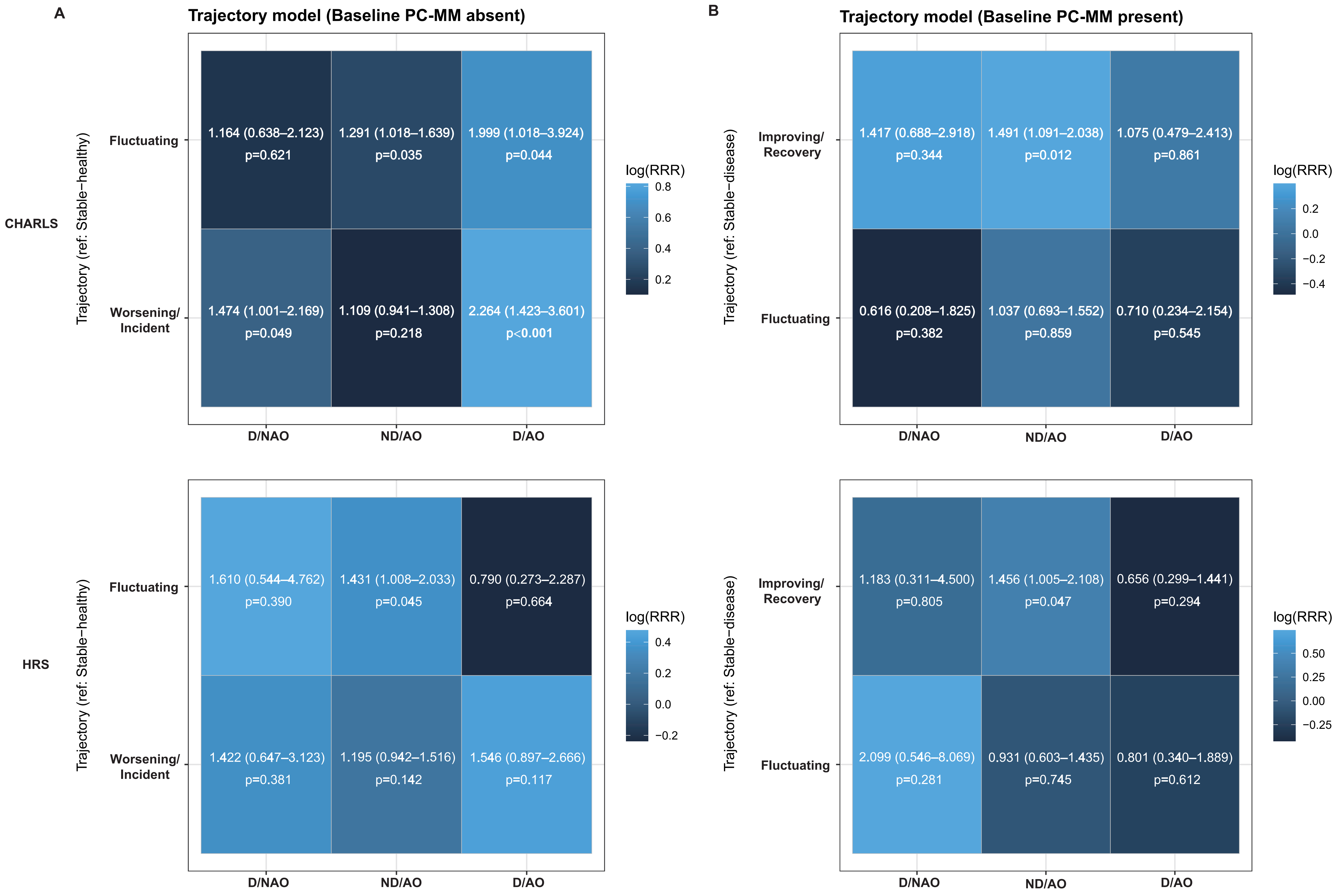
A
Trajectory model (Baseline PC-MM absent)
CHARLS
Trajectory (ref: Stable−healthy)
Fluctuating
Worsening/ Incident
1.164 (0.638–2.123) p=0.621
1.291 (1.018–1.639) p=0.035
1.999 (1.018–3.924) p=0.044
1.474 (1.001–2.169) p=0.049
1.109 (0.941–1.308) p=0.218
2.264 (1.423–3.601) p<0.001
D/NAO
ND/AO
D/AO
log(RRR)
0.8
0.6
0.4
0.2
HRS
Trajectory (ref: Stable−healthy)
Fluctuating
Worsening/ Incident
1.610 (0.544–4.762) p=0.390
1.431 (1.008–2.033) p=0.045
0.790 (0.273–2.287) p=0.664
1.422 (0.647–3.123) p=0.381
1.195 (0.942–1.516) p=0.142
1.546 (0.897–2.666) p=0.117
D/NAO
ND/AO
D/AO
log(RRR)
0.4
0.2
0.0
−0.2
B
Trajectory model (Baseline PC-MM present)
Trajectory (ref: Stable−disease)
Improving/ Recovery
Fluctuating
1.417 (0.688–2.918) p=0.344
1.491 (1.091–2.038) p=0.012
1.075 (0.479–2.413) p=0.861
0.616 (0.208–1.825) p=0.382
1.037 (0.693–1.552) p=0.859
0.710 (0.234–2.154) p=0.545
D/NAO
ND/AO
D/AO
log(RRR)
0.2
0.0
−0.2
−0.4
Trajectory (ref: Stable−disease)
Improving/ Recovery
Fluctuating
1.183 (0.311–4.500) p=0.805
1.456 (1.005–2.108) p=0.047
0.656 (0.299–1.441) p=0.294
2.099 (0.546–8.069) p=0.281
0.931 (0.603–1.435) p=0.745
0.801 (0.340–1.889) p=0.612
D/NAO
ND/AO
D/AO
log(RRR)
0.50
0.25
0.00
−0.25
