# Supplementary figures and images for: Dynapenic abdominal obesity and elevated risk of multidimensional multimorbidity across physical, psychological, and cognitive domains: evidence from longitudinal cohorts
Source: Environ Health Prev Med. 2026 May 23;31:35. doi: 10.1265/ehpm.26-00041 (PMC13222744; doi:10.1265/ehpm.26-00041)

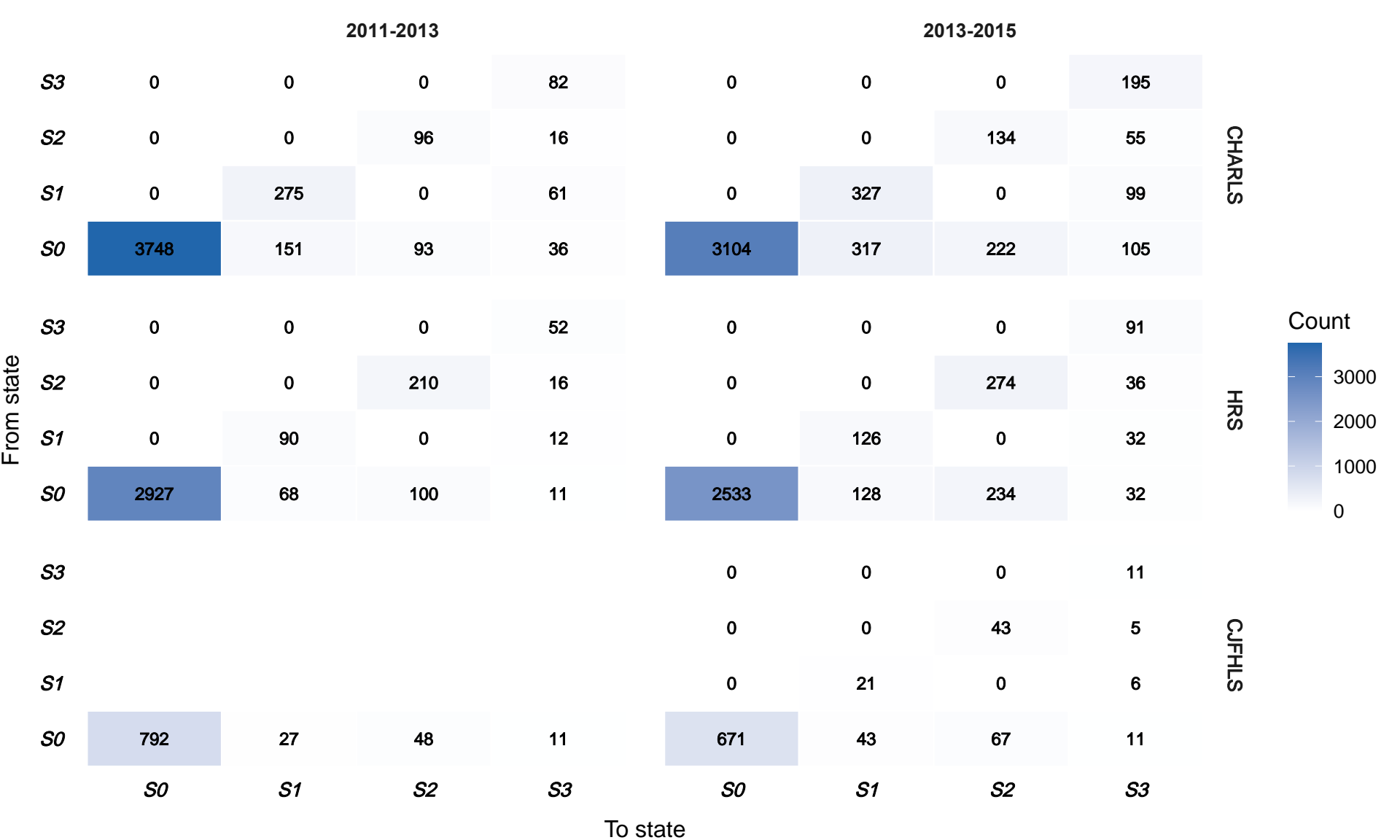

Supplement: Supplementary file 4 — Additional file 4: Supplementary Figure 4. Observed frequency of transitions between mMM in the total analytic sample across adjacent follow-up waves. The matrices display the raw counts of all participants included in the analysis transitioning from a starting state (rows, “From state”) to a subsequent state (columns, “To state”) over specific time intervals (e.g., 2011–2013 and 2013–2015). Color intensity corresponds to the number of participants, with darker blue indicating higher frequencies. Diagonal cells represent individuals who remained in the same state (stability), while off-diagonal cells represent those who transitioned to a different state (worsening or improvement). Health states are defined as: S0, free of multimorbidity; S1, physical-psychological multimorbidity (PP-MM); S2, physical-cognitive multimorbidity (PC-MM); and S3, physical-psychological-cognitive multimorbidity (PPC-MM). Abbreviations: CHARLS, China Health and Retirement Longitudinal Study; HRS, Health and Retirement Study. [file ehpm-31-035-s004.pdf]
