# Supplementary material for: Dynapenic abdominal obesity and elevated risk of multidimensional multimorbidity across physical, psychological, and cognitive domains: evidence from longitudinal cohorts
Source: Environ Health Prev Med. 2026 May 23;31:35. doi: 10.1265/ehpm.26-00041 (PMC13222744; doi:10.1265/ehpm.26-00041)
Supplement: Supplementary file 5 — Additional file 5: Supplementary Materials. [file ehpm-31-035-s005.docx]

**1. China Health and Retirement Longitudinal Study (CHARLS)**

The China Health and Retirement Longitudinal Study (CHARLS) is a nationally representative longitudinal survey hosted by the National School of Development at Peking University. It aims to collect high-quality microdata on households and individuals aged 45 and older in China to support scientific research on aging. The baseline survey was fielded between June 2011 and March 2012, covering 17,708 individuals in 10,257 households.

Sampling Methodology CHARLS employs a strict multi-stage probability-proportional-to-size (PPS) sampling technique to ensure representativeness:

1. County Level: 150 county-level units were randomly selected from a sampling frame of all counties in China (excluding Tibet), stratified by region, urban/rural status, and per capita GDP.
2. Community Level: Within each county, administrative villages or resident committees were selected.
3. Household Level: Households were mapped and randomly sampled within communities.

Data Content Follow-up surveys are conducted biennially using face-to-face Computer-Assisted Personal Interviewing (CAPI). The questionnaire is extensive, covering demographics, family transfer dynamics, health status, health care utilization, work, and household wealth.

A unique feature of CHARLS compared to many other social surveys is its rigorous physical assessment component. In addition to self-reported health data, trained interviewers conduct anthropometric and performance tests, including:

- Biomarkers: Blood samples (collected every two waves).
- Physical Measurements: Height, weight, waist circumference, blood pressure, and lung capacity.
- Functionality Tests: Grip strength, gait speed (walking speed), balance tests, and repeated chair stands.

This rich combination of socioeconomic data and objective health measures allows researchers to analyze the complex challenges of aging in a developing country context.

**2. Health and Retirement Study (HRS)**

Administered by the University of Michigan and sponsored by the National Institute on Aging, the Health and Retirement Study (HRS) is the premier longitudinal panel study serving as a model for aging research globally. Since its inception in 1992, the HRS has collected data on a nationally representative sample of approximately 37,000 adults aged 50 and older across 23,000 households in the United States. The primary objective of the HRS is to capture the dynamic interplay between changing health status and economic circumstances as individuals age, facilitating policy analysis regarding retirement systems, health insurance, and overall well-being.

The survey employs a steady-state design, refreshing the sample every six years with new cohorts to maintain representativeness. The dataset currently comprises several distinct birth cohorts:

- Initial HRS Cohort: Individuals born between 1931 and 1941 (entered in 1992).
- AHEAD Cohort: Those born before 1924 (Asset and Health Dynamics Among the Oldest Old), integrated into the main study after initial separate waves in 1993 and 1995.
- Children of the Depression (CODA): Born 1924–1930 (entered in 1998).
- War Babies (WB): Born 1942–1947 (entered in 1998).
- Early Baby Boomers (EBB): Born 1948–1953 (entered in 2004).
- Mid Baby Boomers (MBB): Born 1954–1959 (entered in 2010).
- Late Baby Boomers (LBB): Born 1960–1965 (entered in 2016).

Data collection occurs biennially. The interview protocol distinguishes between "financial respondents" (knowledgeable about housing, assets, and income) and "family respondents" (knowledgeable about family demographics and transfers) within coupled households, while individual-level health and cognitive questions are answered by all respondents. Due to the complex multi-stage area probability sampling design, analysts must utilize provided sampling weights to correct for selection probabilities and non-response, as well as adjust for stratification and clustering when calculating standard errors.
