# Supplementary material for: Dynapenic abdominal obesity and elevated risk of multidimensional multimorbidity across physical, psychological, and cognitive domains: evidence from longitudinal cohorts
Source: Environ Health Prev Med. 2026 May 23;31:35. doi: 10.1265/ehpm.26-00041 (PMC13222744; doi:10.1265/ehpm.26-00041)
Supplement: Supplementary file 6 — Additional file 6: Supplementary Table 1. Harmonized definitions and measurement strategies for exposures, covariates and outcomes in the present study. [file ehpm-31-035-s006.docx]

**Supplementary Table 1. Harmonized definitions and measurement strategies for exposures, covariates and outcomes in the present study.**

|  | **Harmonized Value** | **Included cohort studies** | |
| --- | --- | --- | --- |
|  |  | **CHARLS** | **HRS** |
| ***Exposure*** | | | |
| *Dynapenia-abdominal obesity status* | *ND/NAO* | maximum hand grip measurement(kg): male>=28, female>=18 & waist measurement in centimeters: male <90, female <85 | maximum hand grip measurement(kg): male>=26, female>=16 & waist measurement (cm): male <=102, female <=88 |
|  | *ND/AO* | maximum hand grip measurement(kg): male>=28, female>=18 & waist measurement in centimeters: male >=90, female >=85 | maximum hand grip measurement(kg): male>=26, female>=16 & waist measurement (cm): male >102, female >88 |
|  | *D/NAO* | maximum hand grip measurement(kg): male<28, female<18 & waist measurement in centimeters: male <90, female <85 | maximum hand grip measurement(kg): male<26, female<16 & waist measurement (cm): male <=102, female <=88 |
|  | *D/AO* | maximum hand grip measurement(kg): male<28, female<18 & waist measurement in centimeters: male >=90, female >=85 | maximum hand grip measurement(kg): male<26, female<16 & waist measurement (cm): male >102, female >88 |
| ***Outcomes*** |  |  |  |
| Physical diseases | Yes | The respondent reported having one or more of the following physician-diagnosed conditions: hypertension, diabetes, stroke, heart disease, arthritis, chronic lung diseases, or cancer. | Defined as having any of the following conditions based on self-reported physician diagnosis and/or current medication use: hypertension (systolic blood pressure >=140 mmHg, diastolic blood pressure >=90 mmHg, history of hypertension, or use of antihypertensive medication), diabetes (history of diabetes or use of glucose-lowering medication), cancer (history of cancer), chronic lung disease (history of lung disease or use of respiratory medication), heart disease (history of heart disease or use of cardiac medication), stroke (history of stroke or use of stroke-related medication), and arthritis (history of arthritis or use of arthritis-related medication). |
|  | No | Did not report any of the physical diseases listed above. | |
| Psychological disorder | Yes (Assessed) | ***CESD-10 score >=***10 | ***CESD-8 score >=***4 |
|  | No (Assessed) | ***CESD-10 score <***10 | ***CESD-8 score <***4 |
|  | Yes (Self-reported) | The respondent reported having affective, emotional, nervous, or psychiatric problems | |
|  | No (Self-reported) | The respondent reported having no affective, emotional, nervous, or psychiatric problems | |
| Cognitive disorder | Yes (Assessed) | Having a Z-score>=1.5 standard deviations below age group-specific means in at least one of four cognitive domains: immediate word recall, delayed word recall, serial 7’s subtraction test, and orientation | *Having a Z-score>=*1.5 standard deviations below age group specific means in at least one of four cognitive domains: immediate word recall, delayed word recall, serial 7’s subtraction test, and orientation |
|  | No (Assessed) | Otherwise | Otherwise |
|  | Yes (Self-reported) | The respondent reported having memory-related problems | The respondent reported having memory-related problems, dementia or Alzheimer’s disease |
|  | No (Self-reported) | The respondent reported having no memory-related problems | The respondent reported having no memory-related problems, dementia or Alzheimer’s disease |
| Physical-psychological multimorbidity(PP-MM) | Yes | Having physical & psychological disorders | |
|  | No | Without physical & psychological disorders | |
| Physical-cognitive multimorbidity(PC-MM) | Yes | Having physical & cognitive disorders | |
|  | No | Without physical & cognitive disorders | |
| Physical-psychological-cognitive multimorbidity (PPC-MM) | Yes | Having physical & psychological & cognitive disorders | |
|  | No | Without physical & psychological & cognitive disorders | |
| **Covariates** | | | |
| Marital status | Married/partnered | What’s marital status: | What’s your legal marital status currently: |
|  |  | 1.married | 1. Married |
|  |  | 2.married, sp abs | 2.Married,spouseabsent |
|  |  | 3.partnered | 3.Partnered |
|  | Other | 4.separated | 4.Separated |
|  |  | 5.divorced | 5.Divorced |
|  |  | 7.widowed | 6.Separated/divorced |
|  |  | 8.never married | 7.Widowed |
|  |  |  | 8.Nevermarried |
| Education level | Low | 1.No formal education illiterate | 1.Lt High-school |
|  |  | 2.Did not finish primary school but capable of reading and/or writing |  |
|  |  | 3.Sishu |  |
|  |  | 4.Elementary school |  |
|  |  | 5.Middle school |  |
|  | High | 6.High school | 2.GED |
|  |  | 7.Vocational school | 3.High-school graduate |
|  |  | 8.Two/Three Year College/Associate degree | 4.Some college |
|  |  | 9.Four Year College/Bachelor's degree | 5.College and above |
|  |  | 10.Post-graduated(Master/PhD) |  |
| Smoking | Non-smoker | smoke ever=0.No & smoke now=0.No | smoke ever=0.No & smokes now=0.No |
|  | Current smoker | smoke ever=1.Yes & smoke now=1.Yes | smokes now=1.Yes |
|  | Ex-smoker | smoke ever=0.NO & smoke now=1.Yes | smoke ever=1.Yes |
| Drinking |  | frequency of drinking last year | ever drinks any alcohol & days/week drinks |
|  | Never | 0.None | ever drinks any alcohol=0.No |
|  | Drink but less than once a week | 1.Less than once a month | Number of days/week that drinks >1 |
|  |  | 2.Once a month |  |
|  |  | 3.2 to 3 days a month |  |
|  |  | 4.Once a week |  |
|  | Drink more than once a week | 5.2 to 3 days a week | Number of days/week that drinks <=1 |
|  |  | 6.4 to 6 days a week |  |
|  |  | 7.Daily |  |
|  |  | 8.Twice a day |  |
|  |  | 9.More than twice a day |  |
| Physical activity |  | Number of days per week with at least one episode of vigorous/moderate physical activity | |
|  | Active | days/wk vigorous physical activity or exer>1 | Freq vigorous phys activ{finerscale}=1.3+perweek |
|  |  | days/wk moderate physical activity or exer>1 | Freq vigorous phys activ{finerscale}=2.1-2perweek |
|  |  |  | Freq moderate phys activ{finerscale}=1.3+perweek |
|  |  |  | Freq moderate phys activ{finerscale}=2.1-2perweek |
|  | Inactive | Otherwise | Otherwise |

Abbreviations: CHARLS, China Health and Retirement Longitudinal Study; HRS, US Health and Retirement Study.
