# Supplementary material for: Dynapenic abdominal obesity and elevated risk of multidimensional multimorbidity across physical, psychological, and cognitive domains: evidence from longitudinal cohorts
Source: Environ Health Prev Med. 2026 May 23;31:35. doi: 10.1265/ehpm.26-00041 (PMC13222744; doi:10.1265/ehpm.26-00041)
Supplement: Supplementary file 7 — Additional file 7: Supplementary Table 2. Trends in multimorbidity risk across different phenotypic progression pathways of dynapenia-abdominal obesity. [file ehpm-31-035-s007.docx]

**Supplementary Table 2.Trends in multimorbidity risk across different phenotypic progression pathways of dynapenia-abdominal obesity.**

| **Cohort** | **Multidimensional Multimorbidity** | | | | | |
| --- | --- | --- | --- | --- | --- | --- |
|  | **PP-MM** | | **PC-MM** | | **PPC-MM** | |
|  | **OR(95%CI)** | **P for trend** | **OR(95%CI)** | **P for trend** | **OR(95%CI)** | **P for trend** |
| **CHARLS** |  |  |  |  |  |  |
| **Model 1** |  |  |  |  |  |  |
| T1(ND/NAO→D/NAO→D/AO) | 1.444(1.148,1.794) | 0.001** | 1.536(1.208,1.925) | <0.001*** | 1.757(1.259,2.368) | <0.001*** |
| T2(ND/NAO→ND/AO→D/AO) | 1.297(1.122,1.499) | <0.001*** | 1.213(1.032,1.425) | 0.019* | 1.344(1.050,1.714) | 0.018* |
| **Model 2** |  |  |  |  |  |  |
| T1(ND/NAO→D/NAO→D/AO) | 1.263(0.991,1.592) | 0.0529 | 1.466(1.136,1.870) | 0.003** | 1.655(1.160,2.295) | 0.004** |
| T2(ND/NAO→ND/AO→D/AO) | 1.167(1.006,1.353) | 0.041* | 1.110(0.940,1.310) | 0.216 | 1.159(0.900,1.490) | 0.25 |
| **Model 3** |  |  |  |  |  |  |
| T1(ND/NAO→D/NAO→D/AO) | 1.266(0.993,1.597) | 0.051 | 1.504(1.164,1.921) | 0.001** | 1.707(1.194,2.372) | 0.002** |
| T2(ND/NAO→ND/AO→D/AO) | 1.174(1.011,1.362) | 0.035* | 1.113(0.941,1.314) | 0.21 | 1.182(0.915,1.521) | 0.197 |
| **HRS** |  |  |  |  |  |  |
| **Model 1** |  |  |  |  |  |  |
| T1(ND/NAO→D/NAO→D/AO) | 1.885(1.366,2.540) | <0.001*** | 1.619(1.234,2.090) | <0.001*** | 2.301(1.433,3.532) | <0.001*** |
| T2(ND/NAO→ND/AO→D/AO) | 1.790(1.383,2.331) | <0.001*** | 1.392(1.132,1.717) | 0.002** | 1.776(1.097,2.922) | 0.022* |
| **Model 2** |  |  |  |  |  |  |
| T1(ND/NAO→D/NAO→D/AO) | 1.676(1.173,2.348) | 0.003** | 1.410(1.035,1.894) | 0.025* | 2.474(1.451,4.088) | 0.001*** |
| T2(ND/NAO→ND/AO→D/AO) | 1.688(1.291,2.219) | <0.001*** | 1.288(1.037,1.606) | 0.023* | 1.733(1.041,2.944) | 0.038* |
| **Model 3** |  |  |  |  |  |  |
| T1(ND/NAO→D/NAO→D/AO) | 1.672(1.160,2.366) | 0.004** | 1.343(0.983,1.811) | 0.058 | 2.524(1.454,4.280) | 0.001*** |
| T2(ND/NAO→ND/AO→D/AO) | 1.643(1.251,2.169) | <0.001*** | 1.230(0.986,1.540) | 0.068 | 1.785(1.077,3.005) | 0.027* |

Data are presented as odds ratios (ORs) [95%CIs] derived from multivariable logistic regression models. Trend tests were conducted by modeling the exposure categories as ordinal variables to assess linear trends across two progression pathways:T1 (ND/NAO → D/NAO → D/AO) and T2 (ND/NAO → ND/AO → D/AO). Model 1 was unadjusted. Model 2 was adjusted for age, sex, marital status, and educational level. Model 3 was additionally adjusted for smoking, alcohol consumption, and physical activity. Abbreviations: CHARLS, China Health and Retirement Longitudinal Study; HRS, Health and Retirement Study; ND/NAO, non-dynapenia and non-abdominal obesity; D/NAO, dynapenia and non-abdominal obesity; ND/AO, non-dynapenia and abdominal obesity; D/AO, dynapenic abdominal obesity; PP-MM, physical-psychological multimorbidity; PC-MM, physical-cognitive multimorbidity; PPC-MM, physical-psychological-cognitive multimorbidity. P < 0.05 was considered statistically significant.
