# Supplementary material for: Dynapenic abdominal obesity and elevated risk of multidimensional multimorbidity across physical, psychological, and cognitive domains: evidence from longitudinal cohorts
Source: Environ Health Prev Med. 2026 May 23;31:35. doi: 10.1265/ehpm.26-00041 (PMC13222744; doi:10.1265/ehpm.26-00041)
Supplement: Supplementary file 8 — Additional file 8: Supplementary Table 3. Modified Poisson regression analyses of dynapenia–abdominal obesity status in relation to multidimensional multimorbidity. [file ehpm-31-035-s008.docx]

**Supplementary Table 3. Modified Poisson regression analyses of dynapenia–abdominal obesity status in relation to multidimensional multimorbidity.**

| **Cohort** | **Model** | **Multidimensional Multimorbidity** | | | | | |
| --- | --- | --- | --- | --- | --- | --- | --- |
|  |  | **PP-MM** | | **PC-MM** | | **PPC-MM** | |
|  |  | **RR(95%CI)** | **P** | **RR(95%CI)** | **P** | **RR(95%CI)** | **P** |
| **CHARLS** | **Model 1** |  |  |  |  |  |  |
|  | ND/NAO | Ref |  | Ref |  | Ref |  |
|  | D/NAO | 1.143 (0.795, 1.644) | 0.471 | 1.312 (0.895, 1.924) | 0.164 | 1.928 (1.112, 3.344) | 0.019* |
|  | ND/AO | 1.187 (1.038, 1.358) | 0.012* | 1.092 (0.934, 1.277) | 0.271 | 1.211 (0.933, 1.572) | 0.15 |
|  | D/AO | 1.997 (1.397, 2.854) | <0.001*** | 2.159 (1.457, 3.201) | <0.001*** | 2.636 (1.389, 5.005) | 0.003** |
|  | **Model 2** |  |  |  |  |  |  |
|  | ND/NAO | Ref |  | Ref |  | Ref |  |
|  | D/NAO | 1.017 (0.699, 1.481) | 0.929 | 1.306 (0.88, 1.94) | 0.185 | 1.957 (1.104, 3.471) | 0.022* |
|  | ND/AO | 1.097 (0.959, 1.255) | 0.177 | 1.009 (0.861, 1.181) | 0.914 | 1.047 (0.806, 1.361) | 0.73 |
|  | D/AO | 1.562 (1.084, 2.249) | 0.017* | 1.936 (1.282, 2.923) | 0.002** | 2.163 (1.105, 4.235) | 0.024* |
|  | **Model 3** |  |  |  |  |  |  |
|  | ND/NAO | Ref |  | Ref |  | Ref |  |
|  | D/NAO | 1.01 (0.692, 1.473) | 0.96 | 1.325 (0.892, 1.969) | 0.164 | 2.001 (1.122, 3.569) | 0.019* |
|  | ND/AO | 1.1 (0.961, 1.26) | 0.166 | 1.009 (0.861, 1.182) | 0.915 | 1.064 (0.818, 1.383) | 0.645 |
|  | D/AO | 1.577 (1.098, 2.264) | 0.014* | 1.978 (1.31, 2.988) | 0.001** | 2.278 (1.166, 4.45) | 0.016* |
| **HRS** | **Model 1** |  |  |  |  |  |  |
|  | ND/NAO | Ref |  | Ref |  | Ref |  |
|  | D/NAO | 1.154 (0.375, 3.551) | 0.803 | 1.826 (0.987, 3.376) | 0.055 | 2.479 (0.592, 10.38) | 0.214 |
|  | ND/AO | 1.614 (1.215, 2.144) | 0.001*** | 1.254 (1.019, 1.544) | 0.032* | 1.233 (0.718, 2.116) | 0.447 |
|  | D/AO | 3.259 (1.931, 5.498) | <0.001*** | 2.176 (1.41, 3.359) | <0.001*** | 4.9 (2.106, 11.403) | <0.001*** |
|  | **Model 2** |  |  |  |  |  |  |
|  | ND/NAO | Ref |  | Ref |  | Ref |  |
|  | D/NAO | 1.071 (0.338, 3.387) | 0.908 | 1.649 (0.895, 3.039) | 0.109 | 2.865 (0.637, 12.877) | 0.17 |
|  | ND/AO | 1.506 (1.131, 2.005) | 0.005** | 1.154 (0.938, 1.42) | 0.176 | 1.13 (0.657, 1.942) | 0.659 |
|  | D/AO | 2.968 (1.735, 5.077) | <0.001*** | 1.917 (1.269, 2.897) | 0.002** | 5.411 (2.358, 12.418) | <0.001*** |
|  | **Model 3** |  |  |  |  |  |  |
|  | ND/NAO | Ref |  | Ref |  | Ref |  |
|  | D/NAO | 1.081 (0.343, 3.413) | 0.894 | 1.679 (0.936, 3.011) | 0.082 | 3.029 (0.71, 12.92) | 0.134 |
|  | ND/AO | 1.489 (1.118, 1.984) | 0.006** | 1.111 (0.9, 1.372) | 0.326 | 1.219 (0.705, 2.108) | 0.478 |
|  | D/AO | 2.636 (1.531, 4.539) | <0.001*** | 1.726 (1.136, 2.622) | 0.01* | 4.677 (2.067, 10.58) | <0.001*** |

Data are presented as relative risks (RRs) and 95% CIs estimated using modified Poisson regression models with robust error variance. Model 1 was unadjusted. Model 2 was adjusted for age, sex, marital status, and educational level. Model 3 was additionally adjusted for smoking, alcohol consumption, and physical activity. Abbreviations: CHARLS, China Health and Retirement Longitudinal Study; HRS, Health and Retirement Study; ND/NAO, non-dynapenia and non-abdominal obesity; D/NAO, dynapenia and non-abdominal obesity; ND/AO, non-dynapenia and abdominal obesity; D/AO, dynapenic abdominal obesity; PP-MM, physical-psychological multimorbidity; PC-MM, physical-cognitive multimorbidity; PPC-MM, physical-psychological-cognitive multimorbidity. P < 0.05 was considered statistically significant.
