# Supplementary material for: Dynapenic abdominal obesity and elevated risk of multidimensional multimorbidity across physical, psychological, and cognitive domains: evidence from longitudinal cohorts
Source: Environ Health Prev Med. 2026 May 23;31:35. doi: 10.1265/ehpm.26-00041 (PMC13222744; doi:10.1265/ehpm.26-00041)
Supplement: Supplementary file 9 — Additional file 9: Supplementary Table 4. Associations of dynapenia–abdominal obesity status with multidimensional multimorbidity under the single-dimension exclusion strategy. [file ehpm-31-035-s009.docx]

**Supplementary Table 4. Associations of dynapenia–abdominal obesity status with multidimensional multimorbidity under the single-dimension exclusion strategy.**

| **Cohort/Model** | **Multidimensional Multimorbidity** | | | | | |
| --- | --- | --- | --- | --- | --- | --- |
|  | **PP-MM** | | **PC-MM** | | **PPC-MM** | |
|  | **OR(95%CI)** | **P** | **OR(95%CI)** | **P** | **OR(95%CI)** | **P** |
| **CHARLS** |  |  |  |  |  |  |
| ND/NAO | Ref |  | Ref |  | Ref |  |
| D/NAO | 0.994 (0.64, 1.497) | 0.977 | 1.385 (0.958, 1.962) | 0.074 | 1.906 (1.207, 2.908) | 0.004** |
| ND/AO | 1.154 (0.994, 1.341) | 0.061 | 1.093 (0.938, 1.274) | 0.255 | 1.12 (0.919, 1.364) | 0.26 |
| D/AO | 1.706 (1.047, 2.711) | 0.027* | 1.64 (1.05, 2.492) | 0.024* | 1.786 (1.019, 2.971) | 0.033* |
| **HRS** |  |  |  |  |  |  |
| ND/NAO | Ref |  | Ref |  | Ref |  |
| D/NAO | 0.958 (0.609, 1.461) | 0.845 | 1.568 (0.997, 2.382) | 0.042* | 1.937 (1.098, 3.225) | 0.016* |
| ND/AO | 1.035 (0.899, 1.191) | 0.633 | 1.062 (0.899, 1.253) | 0.48 | 0.986 (0.794, 1.225) | 0.902 |
| D/AO | 1.885 (1.152, 3.035) | 0.01** | 1.851 (1.071, 3.051) | 0.02* | 1.989 (0.974, 3.705) | 0.042* |

All estimates are derived from multivariable logistic regression models. In this sensitivity analysis, each multimorbidity outcome was evaluated after excluding participants who already had the corresponding multimorbidity dimension at baseline and retaining those with complete follow-up information for that dimension (“single-dimension exclusion” strategy). Abbreviations: ND/NAO, non-dynapenia and non-abdominal obesity; D/NAO, dynapenia and non-abdominal obesity; ND/AO, non-dynapenia and abdominal obesity; D/AO, dynapenic abdominal obesity. PP-MM, physical-psychological multimorbidity; PC-MM, physical-cognitive multimorbidity; PPC-MM, physical-psychological-cognitive multimorbidity.“Ref” denotes the reference category. Asterisks indicate statistical significance.
