# Supplementary material for: Dynapenic abdominal obesity and elevated risk of multidimensional multimorbidity across physical, psychological, and cognitive domains: evidence from longitudinal cohorts
Source: Environ Health Prev Med. 2026 May 23;31:35. doi: 10.1265/ehpm.26-00041 (PMC13222744; doi:10.1265/ehpm.26-00041)
Supplement: Supplementary file 10 — Additional file 10: Supplementary Table 5. Trend analyses of the associations between exposure and multidimensional multimorbidity under the single-dimension exclusion strategy. [file ehpm-31-035-s010.docx]

**Supplementary Table 5. Trend analyses of the associations between exposure and multidimensional multimorbidity under the single-dimension exclusion strategy.**

| **Cohort** | **Multidimensional Multimorbidity** | | | | | |
| --- | --- | --- | --- | --- | --- | --- |
|  | **PP-MM** | | **PC-MM** | | **PPC-MM** | |
|  | **OR(95%CI)** | **P for trend** | **OR(95%CI)** | **P for trend** | **OR(95%CI)** | **P for trend** |
| **CHARLS** |  |  |  |  |  |  |
| T1(ND/NAO→D/NAO→D/AO) | 1.243(0.993,1.541) | 0.052 | 1.313(1.073,1.593) | 0.007** | 1.482(1.158,1.869) | 0.001** |
| T2(ND/NAO→ND/AO→D/AO) | 1.186(1.035,1.359) | 0.014* | 1.135(0.989,1.300) | 0.07 | 1.173(0.983,1.397) | 0.076 |
| **HRS** |  |  |  |  |  |  |
| T1(ND/NAO→D/NAO→D/AO) | 1.234(0.987,1.533) | 0.06 | 1.451(1.139,1.825) | 0.002** | 1.532(1.131,2.026) | 0.004** |
| T2(ND/NAO→ND/AO→D/AO) | 1.097(0.963,1.249) | 0.162 | 1.124(0.965,1.309) | 0.132 | 1.065(0.871,1.301) | 0.538 |

Trend analyses of the associations between dynapenia-abdominal obesity phenotypes and multidimensional multimorbidity across the three cohorts under the “single-dimension exclusion” strategy. Data are odds ratios (ORs) [95% confidence intervals (CIs)] derived from multivariable logistic regression models. Tests for trend (P for trend) were performed by modeling the exposure categories as ordinal variables. Abbreviations: ND/NAO, non-dynapenia and non-abdominal obesity; D/NAO, dynapenia and non-abdominal obesity; ND/AO, non-dynapenia and abdominal obesity; D/AO, dynapenic abdominal obesity. PP-MM, physical-psychological multimorbidity; PC-MM, physical-cognitive multimorbidity; PPC-MM, physical-psychological-cognitive multimorbidity. “Ref” denotes the reference category; asterisks indicate statistical significance.
