# Supplementary material for: Dynapenic abdominal obesity and elevated risk of multidimensional multimorbidity across physical, psychological, and cognitive domains: evidence from longitudinal cohorts
Source: Environ Health Prev Med. 2026 May 23;31:35. doi: 10.1265/ehpm.26-00041 (PMC13222744; doi:10.1265/ehpm.26-00041)
Supplement: Supplementary file 11 — Additional file 11: Supplementary Table 6. Associations of dynapenia–abdominal obesity status with multidimensional multimorbidity after multiple imputation. [file ehpm-31-035-s011.docx]

**Supplementary Table 6. Associations of dynapenia–abdominal obesity status with multidimensional multimorbidity after multiple imputation.**

| **Cohort** | **Multidimensional Multimorbidity** | | | | | |
| --- | --- | --- | --- | --- | --- | --- |
|  | **PP-MM** | | **PC-MM** | | **PPC-MM** | |
|  | **OR(95%CI)** | **P** | **OR(95%CI)** | **P** | **OR(95%CI)** | **P** |
| **CHARLS** |  |  |  |  |  |  |
| ND/NAO | Ref |  | Ref |  | Ref |  |
| D/NAO | 1.017 (0.661, 1.563) | 0.94 | 1.381 (0.883, 2.159) | 0.157 | 2.097 (1.151, 3.819) | 0.016* |
| ND/AO | 1.087 (0.925, 1.276) | 0.31 | 0.986 (0.824, 1.180) | 0.88 | 1.008 (0.764, 1.328) | 0.957 |
| D/AO | 1.867 (1.130, 3.085) | 0.015* | 2.343 (1.381, 3.975) | 0.002** | 2.641 (1.291, 5.403) | 0.008** |
| **HRS** |  |  |  |  |  |  |
| ND/NAO | Ref |  | Ref |  | Ref |  |
| D/NAO | 1.306 (1.003, 1.700) | 0.047* | 1.252 (1.008, 1.554) | 0.042* | 1.142 (0.680, 1.919) | 0.615 |
| ND/AO | 1.153 (0.444, 2.996) | 0.77 | 1.767 (0.898, 3.476) | 0.099 | 2.305 (0.510, 10.412) | 0.277 |
| D/AO | 2.509 (1.414, 4.450) | 0.002** | 2.313 (1.407, 3.803) | <0.001*** | 4.869 (1.993, 11.898) | <0.001*** |

Odds ratios (ORs) with 95% CIs were obtained from multivariable logistic regression models. All analyses were performed on 20 imputed datasets, and pooled estimates were derived using Rubin’s rules. Abbreviations: ND/NAO, non-dynapenia and non-abdominal obesity; D/NAO, dynapenia and non-abdominal obesity; ND/AO, non-dynapenia and abdominal obesity; D/AO, dynapenic abdominal obesity. PP-MM, physical-psychological multimorbidity; PC-MM, physical-cognitive multimorbidity; PPC-MM, physical-psychological-cognitive multimorbidity. “Ref” denotes the reference category.
