# Supplementary material for: Dynapenic abdominal obesity and elevated risk of multidimensional multimorbidity across physical, psychological, and cognitive domains: evidence from longitudinal cohorts
Source: Environ Health Prev Med. 2026 May 23;31:35. doi: 10.1265/ehpm.26-00041 (PMC13222744; doi:10.1265/ehpm.26-00041)
Supplement: Supplementary file 12 — Additional file 12: Supplementary Table 7. Multinomial logistic regression for the associations between dynapenia-abdominal obesity phenotypes and mutually exclusive multidimensional multimorbidity. [file ehpm-31-035-s012.docx]

**Supplementary Table 7. Multinomial logistic regression for the associations between dynapenia-abdominal obesity phenotypes and mutually exclusive multidimensional multimorbidity.**

| **Cohort** |  | **Multidimensional Multimorbidity** | | | | | | | |
| --- | --- | --- | --- | --- | --- | --- | --- | --- | --- |
|  |  | **PP-MM** | |  | **PC-MM** | |  | **PPC-MM** | |
|  |  | **RRR(95%CI)** | **P** |  | **RRR(95%CI)** | **P** |  | **RRR(95%CI)** | **P** |
| **CHARLS** |  |  |  |  |  |  |  |  |  |
| **Model 1** |  |  |  |  |  |  |  |  |  |
| ND/NAO |  | Ref |  |  | Ref |  |  | Ref |  |
| D/NAO |  | 0.814 (0.451,1.469) | 0.495 |  | 0.916 (0.483,1.735) | 0.787 |  | 2.011 (1.058, 3.528) | 0.070 |
| ND/AO |  | 1.196 (0.967,1.479) | 0.099 |  | 1.068 (0.834, 1.369) | 0.601 |  | 1.219 (0.892, 1.665) | 0.214 |
| D/AO |  | 2.117 (1.123,3.988) | 0.020 * |  | 2.164 (1.070, 4.376) | 0.032 * |  | 3.446 (1.634, 7.268) | 0.001** |
| **Model 2** |  |  |  |  |  |  |  |  |  |
| ND/NAO |  | Ref |  |  | Ref |  |  | Ref |  |
| D/NAO |  | 0.676 (0.370,1.233) | 0.201 |  | 1.007 (0.525, 1.932) | 0.983 |  | 1.881 (0.977, 3.620) | 0.059 |
| ND/AO |  | 1.099 (0.883,1.367) | 0.397 |  | 0.992 (0.769, 1.280) | 0.954 |  | 0.991 (0.719, 1.365) | 0.956 |
| D/AO |  | 1.579 (1.825,3.023) | 0.168 |  | 2.352 (1.137, 4.866) | 0.021 * |  | 3.056 (1.398, 6.681) | 0.005** |
| **Model 3** |  |  |  |  |  |  |  |  |  |
| ND/NAO |  | Ref |  |  | Ref |  |  | Ref |  |
| D/NAO |  | 0.665 (0.364,1.214) | 0.184 |  | 1.011 (0.527, 1.941) | 0.973 |  | 1.887 (0.979, 3.638) | 0.058 |
| ND/AO |  | 1.090 (0.875,1.358) | 0.444 |  | 0.980 (0.758, 1.266) | 0.876 |  | 1.003 (0.726, 1.385) | 0.987 |
| D/AO |  | 1.553 (0.811,2.976) | 0.184 |  | 2.347 (1.133, 4.8618) | 0.022* |  | 3.158 (1.442, 6.916) | 0.004** |
| **HRS** |  |  |  |  |  |  |  |  |  |
| **Model 1** |  |  |  |  |  |  |  |  |  |
| ND/NAO |  | Ref |  |  | Ref |  |  | Ref |  |
| D/NAO |  | 0.629 (0.084, 4.708) | 0.652 |  | 1.821 (0.790, 4.200) | 0.160 |  | 2.798 (0.3621, 12.606) | 0.22 |
| ND/AO |  | 1.905 (1.320, 2.749) | 0.001  *** |  | 1.275 (0.988, 1.645) | 0.062 |  | 1.260 (0.713, 2.226) | 0.427 |
| D/AO |  | 3.205 (1.429,7.189) | 0.005** |  | 1.992 (1.032, 3.844) | 0.040* |  | 6.289 (2.508, 15.768) | <0.001 *** |
| **Model 2** |  |  |  |  |  |  |  |  |  |
| ND/NAO |  | Ref |  |  | Ref |  |  | Ref |  |
| D/NAO |  | 0.501 (0.066, 3.793) | 0.504 |  | 1.526 (0.643, 3.623) | 0.338 |  | 3.122 (0.662, 14.715) | 0.150 |
| ND/AO |  | 1.742 (1.198, 2.534) | 0.004** |  | 1.159 (0.889, 1.511) | 0.275 |  | 1.153 (0.641, 2.071) | 0.635 |
| D/AO |  | 2.614 (1.139, 6.001) | 0.023* |  | 1.706 (1.860, 3.382) | 0.126 |  | 7.003 (2.635, 18.610) | <0.001 *** |
| **Model 3** |  |  |  |  |  |  |  |  |  |
| ND/NAO |  | Ref |  |  | Ref |  |  | Ref |  |
| D/NAO |  | 0.500 (0.066, 3.798) | 0.503 |  | 1.558 (0.649, 3.742) | 0.321 |  | 3.358 (0.711, 15.865) | 0.126 |
| ND/AO |  | 1.657 (1.129, 2.433) | 0.01** |  | 1.080 (0.823, 1.416) | 0.580 |  | 1.269 (0.695, 2.316) | 0.438 |
| D/AO |  | 2.300 (0.994, 5.320) | 0.052 |  | 1.509 (0.758, 3.004) | 0.241 |  | 6.532 (2.391, 17.844) | <0.001  *** |

Data are presented as relative risk ratios (RRRs) [95% CIs)] derived from multivariable logistic regression models. Model 1: unadjusted; Model 2: adjusted for age, sex, marital status, and education; Model 3: additionally adjusted for smoking, alcohol consumption, and physical activity. Abbreviations: CHARLS, China Health and Retirement Longitudinal Study; HRS, Health and Retirement Study; ND/NAO, non-dynapenia and non-abdominal obesity; D/NAO, dynapenia and non-abdominal obesity; ND/AO, non-dynapenia and abdominal obesity; D/AO, dynapenic abdominal obesity; PP-MM, physical-psychological multimorbidity; PC-MM, physical-cognitive multimorbidity; PPC-MM, physical-psychological-cognitive multimorbidity; Ref, reference group.
