# Supplementary material for: Dynapenic abdominal obesity and elevated risk of multidimensional multimorbidity across physical, psychological, and cognitive domains: evidence from longitudinal cohorts
Source: Environ Health Prev Med. 2026 May 23;31:35. doi: 10.1265/ehpm.26-00041 (PMC13222744; doi:10.1265/ehpm.26-00041)
Supplement: Supplementary file 13 — Additional file 13: Supplementary Table 8. Pooled associations of dynapenic abdominal obesity status with multidimensional multimorbidity and assessment of cohort heterogeneity. [file ehpm-31-035-s013.docx]

**Supplementary Table 8. Pooled associations of dynapenic abdominal obesity status with multidimensional multimorbidity and assessment of cohort heterogeneity.**

| **Regression Models** | **Model** | **Multidimensional Multimorbidity** | | | | | |
| --- | --- | --- | --- | --- | --- | --- | --- |
|  |  | **PP-MM** | | **PC-MM** | | **PPC-MM** | |
|  |  | **OR(95%CI)** | **P** | **OR(95%CI)** | **P** | **OR(95%CI)** | **P** |
| **Logistic** | ND/NAO | Ref |  | Ref |  | Ref |  |
|  | D/NAO | 0.999 (0.647, 1.492) | 0.997 | 1.389 (0.92, 2.039) | 0.104 | 2.149 (1.168, 3.705) | 0.009** |
|  | ND/AO | 1.204 (1.042, 1.391) | 0.012* | 1.054 (0.91, 1.22) | 0.482 | 1.073 (0.833, 1.382) | 0.586 |
|  | D/AO | 2.149 (1.43, 3.166) | <0.001*** | 2.153 (1.441, 3.157) | <0.001*** | 3.332 (1.81, 5.805) | <0.001*** |
|  | P for interaction | 0.694 | | 0.882 | | 0.63 | |
| **Poission** |  | RR(95%CI) | P | RR(95%CI) | P | RR(95%CI) | P |
|  | ND/NAO | Ref |  | Ref |  | Ref |  |
|  | D/NAO | 1.001 (0.698, 1.434) | 0.997 | 1.323 (0.95, 1.842) | 0.097 | 2.035 (1.185, 3.493) | 0.01** |
|  | ND/AO | 1.17 (1.037, 1.32) | 0.011* | 1.045 (0.921, 1.185) | 0.492 | 1.068 (0.841, 1.357) | 0.589 |
|  | D/AO | 1.832 (1.366, 2.457) | <0.001*** | 1.847 (1.385, 2.464) | <0.001*** | 2.984 (1.808, 4.925) | <0.001*** |
|  | P for interaction | 0.515 | | 0.871 | | 0.529 | |

Logistic regression models were used to estimate odds ratios (ORs) and 95%CIs. Modified Poisson regression models with robust error variance were used to estimate risk ratios (RRs) and 95% CIs. P for interaction represents the statistical significance of the interaction term between dynapenic abdominal obesity status and cohort, indicating whether the magnitude of the association differs significantly across the three cohorts (heterogeneity). Abbreviations: ND/NAO, non-dynapenia and non-abdominal obesity (reference); D/NAO, dynapenia and non-abdominal obesity; ND/AO, non-dynapenia and abdominal obesity; D/AO, dynapenic abdominal obesity; PP-MM, physical-psychological multimorbidity; PC-MM, physical-cognitive multimorbidity; PPC-MM, physical-psychological-cognitive multimorbidity.
